# MolSnapper: Conditioning Diffusion for Structure-Based Drug Design

**DOI:** 10.1021/acs.jcim.4c02008

**Published:** 2025-04-18

**Authors:** Yael Ziv, Fergus Imrie, Brian Marsden, Charlotte M. Deane

**Affiliations:** † Department of Statistics, University of Oxford, St Giles, Oxford OX1 3LB, U.K.; ‡ Nuffield Department of Medicine, University of Oxford, Old Road, Oxford OX3 7BN, U.K.

## Abstract

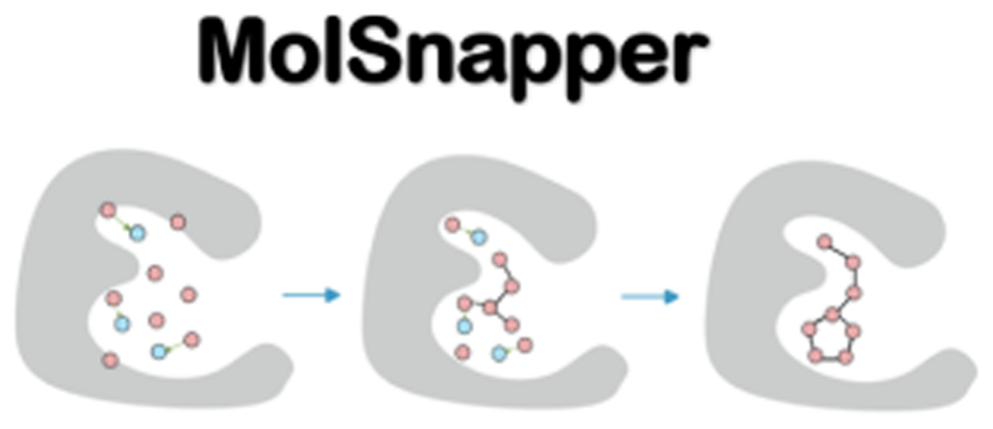

Generative models have emerged as potentially powerful methods for molecular design, yet challenges persist in generating molecules that effectively bind to the intended target. The ability to control the design process and incorporate prior knowledge would be highly beneficial for better tailoring molecules to fit specific binding sites. In this paper, we introduce MolSnapper, a novel tool that is able to condition diffusion models for structure-based drug design by seamlessly integrating expert knowledge in the form of 3D pharmacophores. We demonstrate through comprehensive testing on both the CrossDocked and Binding MOAD data sets that our method generates molecules better tailored to fit a given binding site, achieving high structural and chemical similarity to the original molecules. Additionally, MolSnapper yields approximately twice as many valid molecules as alternative methods.

## Introduction

Drug design is a complex process involving iterative optimization steps to achieve desired biological responses. The vast search space and discontinuous nature of the optimization landscape pose significant challenges, which has led to a reliance on human experts for molecular design. Traditional methods, typically based on trial-and-error approaches, result in high costs and limited productivity.^1,2^

Computational methods have long been central to drug discovery, aiming to reduce costs, expedite processes, and minimize failures.^3^ The emergence of artificial intelligence, especially deep learning, has shown enormous potential to revolutionize the field; it initially found application in property prediction, yielding promising early results.^4,5^ The success of deep learning in this context stems from its flexibility to learn directly from raw data.^6,7^ Another application of deep learning models is compound design. In this area, models generate multiple molecules, aiming to suggest those with desirable properties.^8−10^ One of the most important properties that has been the subject of this type of work is binding of the molecule to its target.^11^

In structure-based drug design (SBDD), the objective is to generate ligands with high affinity and specificity for a specific protein in a specific 3D conformation. However, designing ligands precisely tailored to bind to a target protein remains a persistent challenge. Several machine learning models have been employed in SBDD for molecular design. For example, autoregressive models generate 3D molecules within the target binding site by iteratively adding atoms and bonds.^12−14^ However, a limitation of autoregressive models is the accumulation of errors during the generation process. Additionally, sequential generation methods may not fully capture the complexities of real-world scenarios, as they impose an artificial ordering scheme, potentially losing the global context of the generated ligands.

To address the limitations of autoregressive models, recent studies^9,15−18^ have turned to diffusion models.^19^ These models iteratively denoise data points sampled from a prior distribution to generate samples. Unlike autoregressive models, diffusion model-based methods can simultaneously model local and global interactions between atoms, leading to improved performance.^16,20^

Despite these advances, computational SBDD faces challenges in terms of the synthesizability and chemical feasibility of the generated molecules. The limited volume of experimentally determined structures of protein–ligand complexes often leads models to learn data set biases rather than grasping the true biophysical principles underlying ligand-protein interactions.^21^ Diffusion methods have also been developed and trained on the far larger data set of just molecules which allows better coverage of drug-like space potentially leading to more viable and synthesizable drug candidates.^15^

The practicality of computational SBDD methods is also hindered by an inability to explicitly include expert knowledge, a limitation that becomes particularly evident in the later stages of drug discovery where substantial prior knowledge could and should guide compound design. For deep learning methods to gain widespread adoption, more control over the generative process is essential.^22^

Conditioning, which involves providing additional information or constraints to a model during generation, can be used to achieve such control. Several methods have begun to use expert knowledge to guide compound design, including DEVELOP,^22^ PGMG,^23^ and STRIFE.^24^ These methods employ autoencoder architectures to generate either 1D SMILES strings or 2D graphs of molecules and are trained to integrate target pharmacophores into the generative process, leading to enhanced molecule generation with superior control over the design phase.

Post hoc conditioning of pretrained diffusion models represents another approach to delivering this control. Methods exist that use conditioning for molecule generation in a protein pocket; for example, SILVR^25^ conditions existing diffusion-based models to generate new molecules that fit into a binding site without knowledge of the protein. To achieve this, SILVR introduces a refinement step within the denoising process, employing a linear combination of the generated ligand and the noised reference. However, it requires an equal number of reference and generated atoms, focusing primarily on fragment merging and linker generation. Due to the lack of any knowledge about the protein, the presence of several dummy atoms can lead to clashes of the generated molecules with the protein.^26^

In contrast, DiffSBDD^16^ generates structures using another conditioning method, inpainting. In inpainting, an unconditional diffusion model can generate approximate conditional samples when the context is injected into the sampling process by modifying the probabilistic transition steps. DiffSBDD directly incorporates protein information into the model (rather than in the conditioning process), yet it retains the restriction of using only protein–ligand complex data in its training. Other recent approaches for protein-conditioned generation include PILOT,^27^ a diffusion model that uses importance sampling to optimize generated molecules, and DrugHIVE,^28^ both of which were trained on a combination of unbound molecules and protein–ligand complex data.

Recent studies PoseBusters^29^ and PoseCheck^26^ have highlighted that deep learning methods in SBDD, including diffusion models, frequently produce molecular structures that are physically implausible. This problem arises partly because the models’ performance metrics are not aligned with physical viability. Specifically, deep learning generative models struggle to create the hydrogen bond interactions between the target protein and ligand seen in the ground truth ligands. PoseCheck found that none of the seven methods tested matched or exceeded the ground truth ligand’s interactions with its protein target and that the most common number of hydrogen bond acceptors and donors of the generated molecules was zero.

Moreover, the evaluation of SBDD-generated poses often includes a redocking step, which can mask issues such as steric clashes and elevated strain energies.^26^ For a model to truly be generating physically meaningful structures conditionally on the binding site, the generated molecules and associated poses should be viable without the need for redocking. In particular, docking can significantly shift the molecules from their model-generated positions (for example PoseCheck demonstrated an average RMSD of 0.94 Å to 1.28 Å between the minimized and original poses, with significantly larger RMSDs under full redocking). Thus, to robustly assess the capabilities of SBDD-based generative methods that generate 3D poses, it is important to assess the generated poses directly, with minimal or no further refinement.

In this paper, we propose MolSnapper, a novel tool that conditions diffusion models for SBDD by integrating expert knowledge. Our approach is focused on generating molecules that are not only plausible and valid but also capable of forming hydrogen bonds or other interactions similar to those observed in ground truth ligands. While we utilize physically meaningful 3D structural information, typically provided as 3D pharmacophores,^30^ our framework is not limited to this type of constraint. Recognizing the diverse nature of protein targets, MolSnapper allows users the flexibility to employ various constraint types tailored to their specific protein of interest. These constraints can be manually selected or automatically extracted from experimental data, such as fragment screening experiments.

Protein information is leveraged in the form of user guidance, offering a more adaptable framework for drug design and allowing the utilization of models trained on large molecule data sets for molecule generation. This contrasts with SBDD models that are often trained on limited protein-molecule data.

We benchmarked MolSnapper using the CrossDocked^31^ and Binding MOAD^32^ data sets. In our evaluation, MolSnapper outperformed SILVR, which, similarly to our approach, operates without additional training on protein–ligand data. MolSnapper also achieved results comparable to data set-specific conditioning methods trained on specific protein–ligand data. Our results demonstrate the efficacy of MolSnapper in improving the proportion of generated molecules that closely resemble the reference molecule. Furthermore, our approach is able to generate 3D molecular structures that fulfill the desired constraints and successfully reproduce the majority of hydrogen bond interactions observed between the reference ligand and the protein, and yields approximately twice as many valid molecules as other methods.

## Methods

We create a generative diffusion model to predict molecules for a given protein pocket that takes advantage of the large available training data in drug-like molecule space and integrates physically meaningful 3D structural information into the generative process using 3D pharmacophores.^30^

As our base model, we use MolDiff^15^ as it is among the top-performing models for molecule generation.^15^ MolDiff was pretrained on the GEOM-Drug data set.^33^ We do not retrain MolDiff; instead, we condition it without altering the model weights.

A 3D small molecule is characterized by atom types, atom positions (coordinates in space), and bond types. A molecule with *N* atoms can be denoted as *M* = {*A*, *R*, *B*}, where *A* = {*a*_*i*_}_*N*_ ∈ **A**^*N*^ represents atom types, *R* = {*r*_*i*_}_*N*_ ∈ **R**^*N* × 3^ represents atom positions, and *B* = {*b*_*ij*_}_*N* × *N*_ ∈ **B**^*N* × *N*^ represents chemical bonds Additionally, specific positions related to pharmacophores are denoted as , representing the fixed positions and types of the pharmacophores, and , representing the fixed atom positions associated with pharmacophores, and , representing the positions of the target protein.

In the context of the diffusion model framework, following the framework introduced in the MolDiff paper,^15^ during the reverse process, the Markov chain is reversed to reconstruct the true sample. This involves using *E*(3)-equivariant neural networks to parametrize the transition *p*_θ_(*M*^*t*–1^|*M*^*t*^) from prior distributions, where  and . Specifically, the predicted atom positions are modeled as a Gaussian distribution , and the atom types are modeled as categorical distributions  where μ_θ_ and *H*_θ_ are neural networks. Here, Σ_*t*_ is set as β_*t*_ where β_*t*_, *t* ∈ [0, 1] is the predefined noise scaling schedule.

In our approach, the unconstrained diffusion process is altered in that the parametrized distribution is conditioned by the pharmacophores and the protein positions. We modify the reverse process such that  and , while the bonds prediction remains unchanged.

### Pharmacophores

Pharmacophores can be used as a representation of chemical interactions crucial for ligand binding to macromolecular targets.^30^ These interactions include hydrogen bonds, charge interactions, and lipophilic contacts. Pharmacophores can be derived through ligand-based or structure-based approaches, providing versatility in their application. In this study, we employed 3D pharmacophores derived from ground truth molecules, using the 3D positions and types (donor or acceptor) of these pharmacophores to condition the reverse generative process.

Given their widespread relevance in drug discovery, our chosen pharmacophores included hydrogen bond donor and acceptor features, determined according to default RDKit definitions. Hydrogen bonds both play an important role in various protein–ligand interactions^34^ and generative models, as highlighted by PoseCheck,^26^ often face challenges in reproducing hydrogen bond interactions compared to the ground truth ligand.

While our framework incorporates specific constraints in this study, it is inherently flexible and can be extended to include additional constraints based on user preferences. Users can tailor the features to guide the design process according to their specific requirements.

### Constrain Positions

To constrain the reverse generation of atom positions, a mask *X* ∈ {0, 1} is introduced, where Mask *X* is applied to limit the modification to the positions of the pharmacophores. The predefined noise scaling schedule β^*t*^ is incorporated:
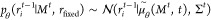
1where
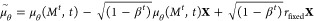
2

At each stage of the process, we compel certain atom positions to approach the fixed locations. In the early steps, we facilitate the atoms to move slightly toward the fixed positions, while in the final steps, we firmly anchor them in place.

**Figure 1:**
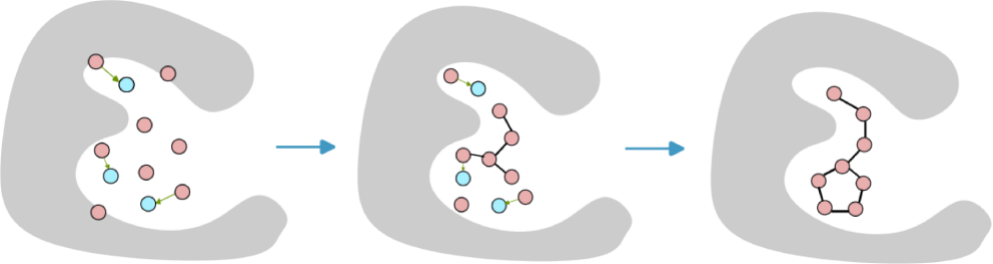
Visualization of the sampling process depicting atom positions constrained by fixed locations. The pink spheres represent the positions of the generated molecule, the blue spheres indicate the positions of the fixed reference points, and the gray mesh represents the protein surface. At each step after the diffusion outputs the position at time *t*, some atoms are gradually moved toward the fixed positions, aiding in the refinement of the molecular structure.

Unlike other methods, we adopt the strategy of utilizing the original positions of the pharmacophores reference points without introducing additional noise. This decision is grounded in the observation that in diffusion models for molecule generation, we operate the reverse process in the same space as the molecule space. Our empirical findings indicate that this approach yields comparable or even superior results compared to incorporating additional noise (see Results and Discussion—Ablation Studies). Furthermore, rather than opting for random initialization, we initialize the positions randomly but around the pharmacophore positions. In Figure 1, we illustrate sampling of positions constrained by fixed positions.

### Guidance Preventing Clashes

Since the MolDiff model was not trained with protein data, it cannot process pocket information. Therefore, it is crucial to incorporate guidance to prevent clashes or excessively short distances between the generated ligand and the protein. As proposed by Sverrisson et al.^35^ and Guan et al.^20^ we describe the protein’s surface as
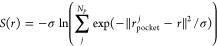
3where  represent the protein atoms’ positions. Following, Guan et al.^20^ the clash guidance loss function is defined as
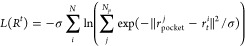
4

**Figure 2:**
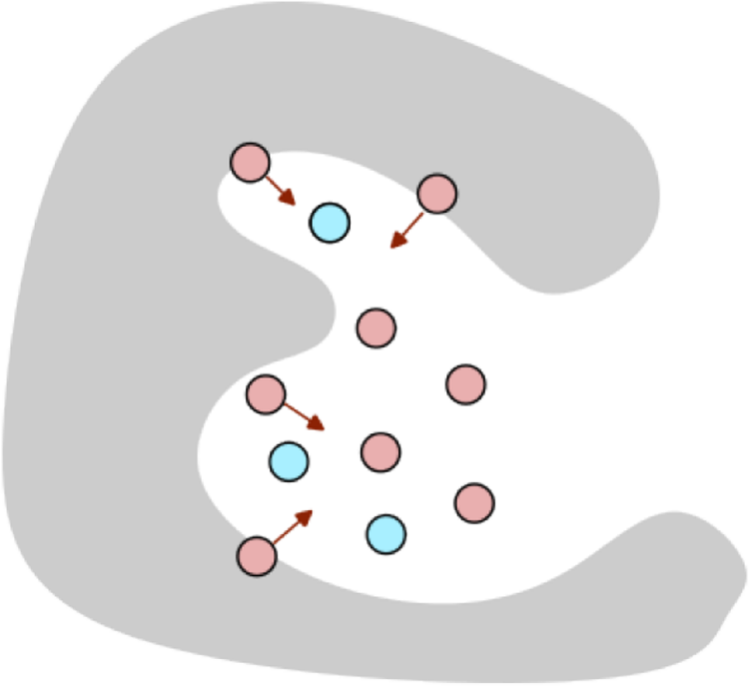
Illustration of clash guidance in the molecule generation process. The fixed positions are shown in blue, the pink positions represent the generated molecule, and the gray mesh represents the protein surface. Clash guidance, inspired by, Guan et al.^20^ prevents clashes or excessively short distances between the generated ligand and the protein. The gradient of the clash guidance loss, denoted as ∇*L*(*R*^*t*^), provides the direction for enhancing molecule generation.

Then the gradient of *L*(*R*^*t*^) with respect to *R*^*t*^, (∇*L*(*R*^*t*^)), provides the direction to enhance molecule generation. With the guidance, the reverse generation of the atom positions, given by
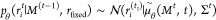
5can be adjusted as
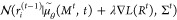
6where λ > 0 is a constant coefficient controlling the strength of the guidance. The clash guidance is illustrated in Figure 2.

### Constrain Atom Types

To enhance interpretability and control over the generated molecules, we allow users to constrain atom types based on their specific requirements. Users can specify the desired atom types in the reference, such as based on the interactions they wish to mimic or the reference structure they intend to use. Throughout the iterative sampling process, the model consistently applies these user-defined constraints, ensuring that the generated molecules align with the intended design objectives. This approach provides flexibility for domain experts to modify this choice as needed in the final output.

Our method introduces a hyperparameter representing the Pharmacophore Satisfaction threshold, evaluating whether the ligand fulfilled the 3D pharmacophore constraints (distance lower than 1 Å and matching family type). We kept only those ligands meeting this criterion across three attempts.

### Experimental Setup

#### Data Sets

We evaluate the efficacy of our conditioning method on two data sets: the CrossDocked2020 data set,^31^ which was constructed by redocking and cross-docking known binders with experimental structures, and the Binding MOAD data set,^32^ which consists exclusively of experimentally determined protein–ligand complexes.

##### CrossDocked2020^31^

For the CrossDocked2020 data set, we followed the same filtering and splitting strategies as in previous work,^12,14^ where only high-quality docking poses (RMSD < 1 Å) and diverse proteins (sequence identity using MMseqs2^36^ < 30%) were retained. This resulted in 100,000 high-quality protein–ligand pairs for the training set and 100 proteins for the test set.

We ran the test set through PoseBusters^29^ and used the Open Drug Discovery Toolkit^37^ (ODDT) to check for the existence of hydrogen bond interactions between the ligand and the protein. To ensure that the test set was of high quality, we kept only those that passed all PoseBusters tests. Further, so that each test case contained relevant pharmacophores, we selected only examples that had more than three hydrogen bond interactions.

This process yielded a set of 73 complexes from the initial 100. For a full list of these complexes, please refer to Section S1. When calculating the success rate (see Evaluation), we further filtered the remaining 73 complexes using the NIH filter^38,39^ resulting in a final set of 48 complexes to evaluate the “success rate”.

##### Binding MOAD^32^

We filtered and categorized complexes based on the proteins’ enzyme commission number, following the approach by Schneuing et al.^16^ This yields 40,344 protein–ligand pairs for training and 130 pairs for testing.

Similar to the CrossDocked2020 data set, our selection criteria for the test set involved passing all PoseBusters tests^29^ and having more than 3 hydrogen bonds. This yielded a set of 43 complexes from the initial 130. For a detailed list of these complexes, please refer to Section S2.

#### Comparison to Other Methods

We compared MolSnapper against other methods designed to condition ligand generation within a protein pocket.

##### MolDiff^15^

To validate the effectiveness of our conditioning, we compared it to MolDiff, without conditioning. Following molecule generation, the ligands generated by MolDiff were docked using the Vina method^40^ as molecules generated by MolDiff are not built in the pocket.

##### SILVR^25^

It is a method tailored for generating molecules to fit into protein binding sites. We utilized their code, specifying pharmacophore positions and atom types as references, treating the remaining atoms as dummy atoms. The SILVR rate was set to 0.01, following the original paper.

##### DiffSBDD^16^

Employs inpainting, a conditioning method for flexible molecule design. For DiffSBDD, we provided pharmacophore positions and atom types as input for the fixed atoms.

In all experiments, we generated 100 new ligands for each reference, allowing three attempts for each generation.

#### Evaluation

##### PoseBusters Pass Rate (Pass Rate)

This metric indicates the percentage of generated molecules that pass PoseBusters.^29^ Our evaluation focuses on ligands that pass the PoseBusters tests. In order to ensure that we only consider ligands that are physically plausible. The PoseBusters test suite examines aspects of chemical validity and consistency, intramolecular validity, and intermolecular validity, assessing physicochemical consistency and structural plausibility in the generated poses.

##### Shape and Color Smilarity Score (SC_RDKit_)

Assesses the 3D similarity between generated molecules and a reference molecule, as described in Imrie et al.^22^ SC_RDKit_ scores range from 0 (no match) to 1 (perfect match). The color similarity function evaluates the overlap of pharmacophoric features, while the shape similarity measure involves a simple volumetric comparison between the two conformers. SC_RDKit_ uses two RDKit functions, based on the methods described in Putta et al.^41^ and Landrum et al.^42^

##### Synthetic Accessibility (SA)

Several scores exist that estimate the synthetic accessibility of a compound.^43−45^ We used SAscore,^43^ which assesses compounds using historical data from synthesized chemicals to estimate synthetic knowledge and molecule complexity. High SA scores denote compounds that are simpler to synthesize, favored in drug development, while low SA scores highlight potential synthetic challenges.^43^

##### Interaction Similarity

This metric calculates the percentage of hydrogen bonds shared between the generated ligands and the protein, and the reference ligand and the protein, using the Open Drug Discovery Toolkit^37^ (ODDT).

##### Molecular Diversity

We assess the diversity of the generated molecules using pairwise Tanimoto similarities of Morgan fingerprints (radius 2, 2048 bits). Diversity is defined as 1-average pairwise similarity, and thus ranges from 0 to 1.

##### Docking Score

We used AutoDock Vina^40^ to further assess the quality of the generated molecules. We used the Vina scoring function to assess molecules with binding site conditional poses and docked molecules that were not generated with a pose conditioned on the binding site. Since such scoring functions are sensitive to the exact coordinates and can assign experimental structures poor scores without energy minimization, we computed energy-minimized scores for generated molecules with binding site conditional poses.^28^ Further, since docking scores are highly correlated with molecular size, we additionally consider normalized docking scores by dividing the score by the square root of the number of atoms.^27^ See Section S3.1 for further details of the exact docking protocol.

##### Success Rate

This metric evaluates the proportion of molecules that not only pass the initial filtering stages but also are considered more likely to be viable drug candidates. First, we exclude molecules flagged by the NIH^38,39^ filter from the test set to remove potentially problematic structures. The NIH filter, implemented using RDKit, screens out substructures known for undesirable properties such as reactive functionalities and medicinal chemistry exclusions. We then apply thresholds for SA and QED based on the minimum values found in the test set. We report the mean number of generated molecules that, after passing PoseBusters, also passed this filtering, in addition to reporting the number of pockets with no ligands that passed these filters (“# Empty Pockets”).

As part of our evaluation strategy, we employed a focused approach centered on SC_RDKit_ scores. For each metric (SA and interaction similarity), we first identify the subset of ligands with the best SC_RDKit_ scores—“Top 1” being the single best, and ‘Top 3′ encompassing the three best-scoring ligands. Once these subsets are determined based on their SC_RDKit_ scores, we then calculate the other metrics specifically for these groups. For example, when we refer to “Top 3 SA,” this is the best Synthetic Accessibility score obtained from the set of the top three ligands as ranked by their SC_RDKit_ scores. Additionally, following common practice, we excluded water molecules from the generation and evaluation process. By including further analysis on the top SC_RDKit_ performers, we aim to highlight those candidates most likely to succeed in real-world drug development scenarios.

## Results and Discussion

### Conditioning Methods without Pocket-Specific Training

In Table 1, we compare MolSnapper to MolDiff without any conditioning and to SILVR, a conditioning method that, like our approach, was trained on a molecule data set alone rather than on protein–molecule data. On the CrossDocked data set, SILVR only generated at least three molecules (out of 100 attempts) that passed the PoseBusters checks for 68 out of 73 complexes. Table 1 shows the results for these 68 complexes. The results for the full 73 complexes for MolSnapper and MolDiff can be found in Table S1. The “success rate” metric was determined using QED and SA thresholds of 0.19 and 0.33, respectively, based on the minimum values observed in the test set.

**Table 1:** Comparison on the CrossDocked Data Set of MolDiff (without Conditioning), SILVR, and Our Method, MolSnapper^a^^b^

	MolDiff (w/o conditioning)	SILVR	MolSnapper
Pass Rate ↑	**91% ± 15%**	27% ± 15%	58% ± 22%
SC_RDKit_ Top 1 ↑	0.417 ± 0.111	0.586 ± 0.148	**0.721 ± 0.116**
SC_RDKit_ All ↑	0.245 ± 0.074	0.416 ± 0.117	**0.576 ± 0.108**
SA Top 1↑	**0.866 ± 0.100**	0.543 ± 0.089	0.631 ± 0.184
SA Top 3↑	**0.907 ± 0.065**	0.610 ± 0.079	0.696 ± 0.163
Interaction Sim. Top 1 ↑	0.276 ± 0.217	0.493 ± 0.273	**0.746 ± 0.249**
Interaction Sim. Top 3 ↑	0.426 ± 0.254	0.654 ± 0.269	**0.810 ± 0.207**
Diversity Top 3↑	0.807 ± 0.117	**0.902 ± 0.045**	0.722 ± 0.245
Normalized Vina Top 1↓	**–1.495 ± 0.732**	–1.081 ± 0.319	–1.176 ± 0.915
Normalized Vina Top 3↓	**–1.616 ± 0.741**	–1.197 ± 0.276	–1.350 ± 0.455
Normalized Vina All↓	**–1.223 ± 1.440**	–1.013 ± 0.383	–1.197 ± 0.412
Success Rate↑	86.68% ± 12.36%	7.05% ± 4.75%	45.00% ± 24.35%
# Empty Pockets↓	0	2	2

a Arrows next to metrics indicate superiority (up: larger is better, down: smaller is better).

b The best results are highlighted in bold. Metrics are defined in Methods—Experimental Setup—Evaluation.

Table 1 shows that MolDiff (the unconditional base model) produced ligands that have high Synthetic Accessibility (SA) scores (average 0.866 for Top 1). Since poses generated by MolDiff are not conditional on the pocket, to check how these molecules may interact with the protein, we docked them into the protein pocket (see Methods for further details). The docked ligands tend to pass the PoseBusters tests (91% PoseBusters pass rate) and have a high success rate of 86.68%. However, in general, the ligands generated by MolDiff failed to recapitulate the interactions shown by the reference ligand (average Top 1 Interaction Similarity 0.276).

SILVR and MolSnapper generate ligands conditioned on the 3D pharmacophore positions, positioned within the binding site. Both methods generate molecules with lower average SA scores compared to unconditioned MolDiff, with the MolSnapper outputs being more synthesizable (SILVR average Top 1 SA 0.543 vs MolSnapper 0.631). SILVR and MolSnapper also show lower PoseBusters Pass Rates than original MolDiff; however, 58% of molecules generated by MolSnapper pass compared to only 27% for SILVR. Furthermore, MolSnapper demonstrates a higher success rate, achieving 45% compared to 7% on average for SILVR.

The SC_RDKit_ scores of the MolSnapper-generated molecules (Top 1 average 0.721) are significantly higher than those from MolDiff or SILVR (Top 1 average 0.417 and 0.586, respectively). Moreover, both SILVR and MolSnapper, due to their conditioning approach, better regenerate the original hydrogen bonds compared to MolDiff. MolSnapper outperforms SILVR, with an average Top 1 “Interaction Similarity” of 0.746 compared to 0.493 for SILVR. Furthermore, we find that MolSnapper generates molecules with improved normalized Vina docking scores compared to SILVR. Perhaps surprisingly, the molecules generated by MolDiff have the best docking scores. We note that we had to dock the MolDiff molecules, rather than only performing energy minimization, which generally results in better docking scores. Additionally, the Vina score is known to only be weakly correlated with binding affinity^31^ and we observe that the docking scores do not correlate with other metrics, such as SC_RDKit_ or Interaction Recovery.

These results demonstrate that MolSnapper consistently outperforms SILVR across all metrics and improves the ability to generate molecules with specific 3D features compared to MolDiff. Without redocking, MolSnapper achieves a 58% PoseBusters Pass Rate, high similarity to the original ligand, and the regeneration of most existing hydrogen bond interactions. The importance of these aspects lies in the preservation of crucial interactions and similarity to the real ligand, which are pivotal for maintaining the pharmacological relevance and effectiveness of the generated molecules.

The differences in performance with SILVR can be attributed to several issues observed with the generated molecules. One issue is the presence of numerous disconnected fragments in SILVR’s generated molecules. In our evaluation, we addressed this by selecting the largest fragment generated. Additionally, clashes with the protein were observed due to SILVR’s model not incorporating protein information. This is further compounded by the fact that our base model, MolDiff,^15^ surpasses the base model that was used for SILVR, EDM,^9^ primarily because it models and diffuses the bonds of the molecule, resulting in the generation of molecules with better validity and Synthetic Accessibility.^15^

**Figure 3:**
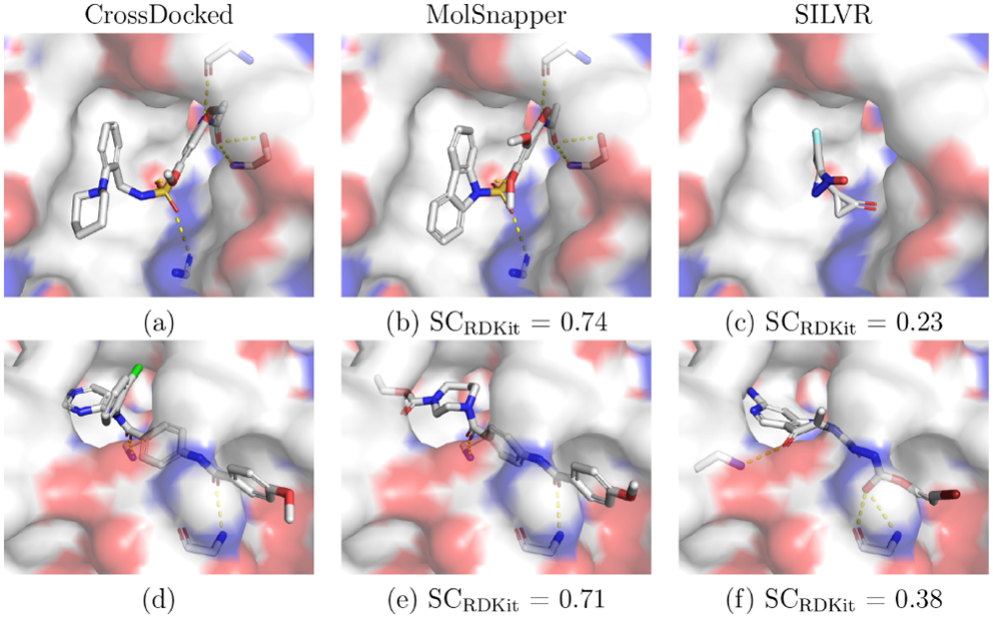
Examples from the CrossDocked data set and molecules generated by SILVR and MolSnapper. (a) 4aaw_A_rec_4ac3_r83_lig_tt_min_0, (d) 5aeh_A_rec_5aeh_8ir_lig_tt_docked_0 are shown with ligands generated by (b), (e) MolSnapper, and (c), (f) SILVR, together with the corresponding SC_RDKit_ scores. Protein surfaces are colored by electrostatic potential: red for acidic, blue for basic, and white for neutral. Hydrogen bonds are depicted as dashed yellow lines.

Figure 3 provides examples of generated molecules from SILVR and MolSnapper, focusing on the best SC_RDKit_-scoring ligands. In the first example (Figure 3c), SILVR struggles to generate a molecule satisfying the given pharmacophore constraint, with the relatively small fragment unable to form a hydrogen bond with the protein. In the second example (Figure 3f), a molecule of an appropriate size is generated, but it fails to form the same bonds as the reference ligand.

### Conditioning Methods with Pocket-Specific Training

We also benchmarked our method against the inpainting conditioning approach employed by DiffSBDD,^16^ a 3D-conditional diffusion model specifically trained on protein–ligand complex data. In our comparison, we evaluated MolSnapper against DiffSBDD trained on CrossDocked data using the CrossDocked data set, where the “success rate” metric was determined using QED and SA thresholds set at 0.19 and 0.33. Additionally, to ensure a more comprehensive assessment that better simulates real-world drug discovery challenges, we extended our analysis to a more demanding data set aligning with actual drug discovery-the Binding MOAD data set. Here, we compared MolSnapper to DiffSBDD trained on Binding MOAD where the QED and SA thresholds were set at 0.22 and 0.31.

Table 2 illustrates that DiffSBDD and MolSnapper show comparable performance in generating molecules that are similar to the original molecule (“SC_RDKit_”) and in recapitulating the binding interactions (“Interaction Sim.”). The molecules generated by DiffSBDD generally have more favorable docking scores than MolSnapper on BindingMOAD, but have worse scores on average on the CrossDocked data set. However, unlike DiffSBDD, MolSnapper does not require a data set of complexes for training, using the same model for both test sets. Additionally, MolSnapper produces both more physically viable and drug-like molecules (“PoseBusters Pass Rate” and “Success Rate”) and more synthetically accessible ones (“SA score”). These results indicate that training on the larger molecule space and conditioning for pocket generation, rather than training only on the protein-molecule set, gives a wider and, therefore, better representation of real molecules.

**Table 2:** Comparison on the CrossDocked Data Set between DiffSBDD (Trained on CrossDocked) and Our Method, MolSnapper, and on Binding MOAD between DiffSBDD (Trained on Binding MOAD) and MolSnapper^a^^b^

	CrossDocked		Binding MOAD	
	DiffSBDD (CrossDocked)	MolSnapper	DiffSBDD (Binding MOAD)	MolSnapper
Pass Rate ↑	47% ± 19%	**58% ± 22%**	31% ± 16%	**57% ± 18%**
SC_RDKit_ Top 1 ↑	0.628 ± 0.141	**0.714 ± 0.116**	**0.557 ± 0.146**	0.537 ± 0.075
SC_RDKit_ All ↑	0.454 ± 0.131	**0.571 ± 0.108**	0.363 ± 0.105	**0.435 ± 0.058**
SA Top 1 ↑	0.638 ± 0.118	**0.642 ± 0.182**	0.545 ± 0.125	**0.706 ± 0.109**
SA Top 3 ↑	0.676 ± 0.104	**0.706 ± 0.162**	0.601 ± 0.093	**0.784 ± 0.076**
Interaction Sim. Top 1 ↑	**0.783 ± 0.219**	0.746 ± 0.246	0.839 ± 0.321	**0.937 ± 0.221**
Interaction Sim. Top 3 ↑	**0.857 ± 0.187**	0.816 ± 0.206	0.938 ± 0.182	**0.968 ± 0.160**
Diversity Top 3 ↑	**0.752 ± 0.220**	0.728 ± 0.238	**0.868 ± 0.035**	0.822 ± 0.053
Normalized Vina Top 1 ↓	**–1.322 ± 0.333**	**–**1.212 ± 0.898	**–1.144 ± 0.357**	**–**0.949 ± 0.391
Normalized Vina Top 3 ↓	**–1.448 ± 0.353**	**–**1.375 ± 0.459	**–1.251 ± 0.326**	**–**1.077 ± 0.430
Normalized Vina All ↓	**–**1.185 ± 0.567	**–1.222 ± 0.413**	**–1.068 ± 0.282**	**–**0.954 ± 0.384
Success Rate ↑	24.6% ± 19.13%	**44.58% ± 24.28%**	13.26% ± 8.05%	**33.56% ± 21.99%**
# Empty Pockets ↓	1	2	0	0

a Arrows next to metrics indicate superiority (up: larger is better, down: smaller is better).

b The best results are highlighted in bold. Metrics are defined in Methods—Experimental Setup—Evaluation.

### Structure-Based Drug Design for a Specific Target

In this section, we further demonstrate the applicability of MolSnapper through two case studies from the literature. These cases involve drug development where either a shared pharmacophore with inhibitors was targeted or there was a need to mimic interactions known from existing inhibitors. The second case highlights MolSnapper’s capability for scaffold hopping, showing that it can be effectively used not just for generating whole molecules, but also for modifying and expanding existing scaffolds to achieve desired interactions.

#### Case Study 1: De Novo Design with Pharmacophore Constraints

Yan et al.^46^ investigated the development of metallo-β-lactamase (MBL) inhibitors. β-Lactam antibiotics, known for disrupting the integrity of bacterial cell walls, are among the most widely used drugs for treating bacterial infections. However, their efficacy is increasingly compromised by bacterial resistance mechanisms, including the production of serine-β-lactamases (SBLs) and metallo-β-lactamases. Yan et al. designed 2-aminothiazole-4-carboxylic acids (AtCs) (e.g., PDB IDs: 8HX5, 8HYD) to mimic the binding interactions of carbapenem hydrolyzate with MBLs (PDB ID: 6Y6J). By introducing 2-aminothiazole-4-carboxylic acid as a core scaffold, they conducted thorough investigations to enhance potency across multiple MBL subclasses, closely mimicking the essential binding features of known MBL inhibitors, although the binding positions were not fully aligned with those of the reference inhibitor.

**Figure 4:**
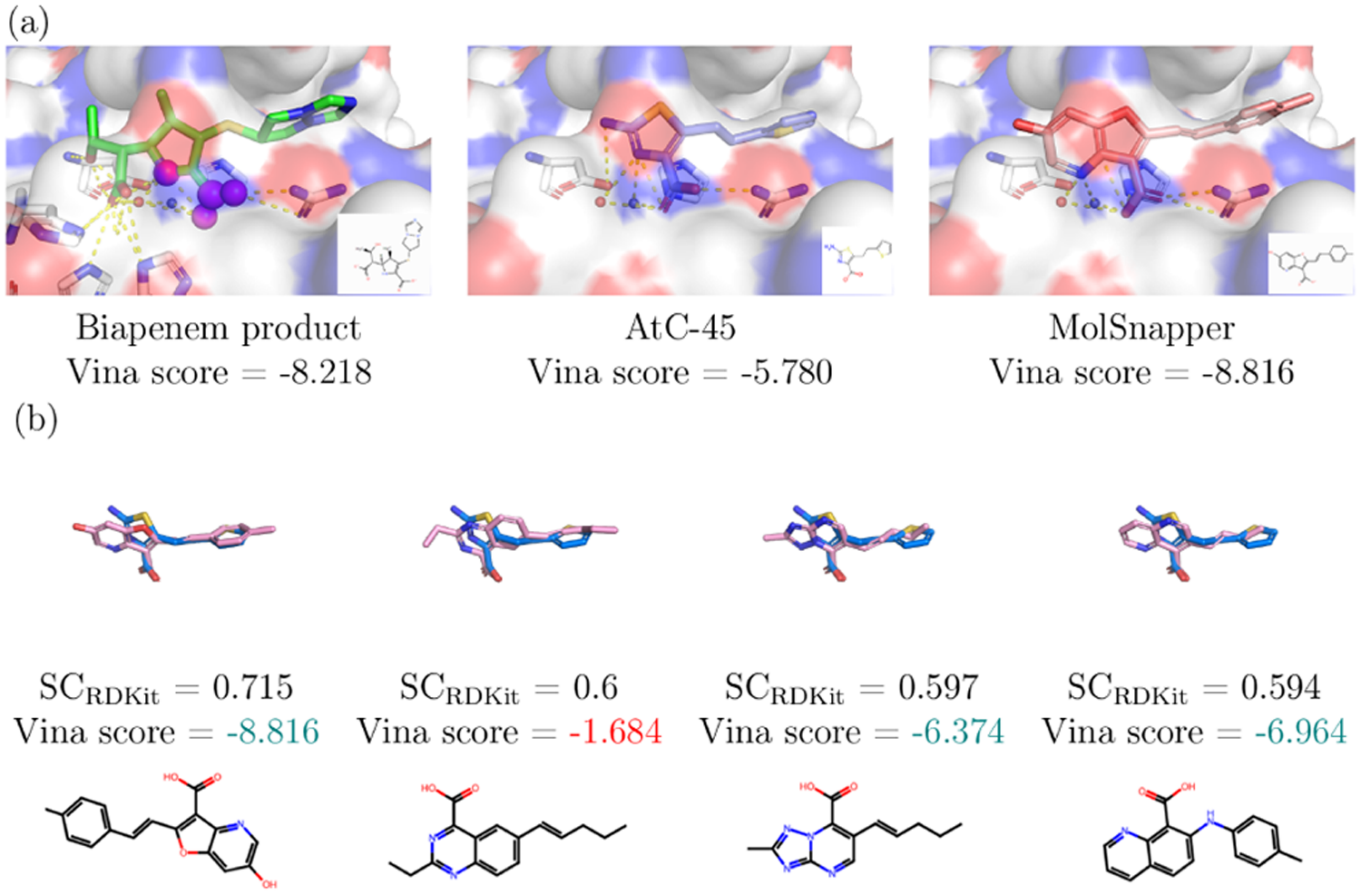
Case Study 1: De Novo Design with Pharmacophore Constraints. (a) Left: the biapenem product (PDB ID: 6Y6J) in complex with VIM-2, with magenta spheres indicating the desired interactions, Middle: the compound developed by Yan et al.^46^ (AtC-45), Right: the molecule generated by MolSnapper with the highest SC_RDKit_ score. Dashed yellow lines indicate the interactions between VIM-2 and the ligand. (b) The molecules generated by MolSnapper with the highest SC_RDKit_ scores (pink) together with AtC-45 developed by Yan et al.^46^ (blue). The molecules are ordered by decreasing SC_RDKit_ score. Vina scores are shown in green if better than AtC-45 and in red if worse.

To demonstrate the application of MolSnapper for de novo design guided by known pharmacophores, we generated molecules using several pharmacophoric features exhibited by the biapenem product, without any further constraints (Figure 4). In particular, we sought to retain the carboxyl group and the hydrogen bond donor forming an interaction with the aspartic acid residue (ASP 118). This closely follows the design goal targeted by Yan et al.^46^ We generated 5000 molecules using MolSnapper and applied a series of filters, including QED, SA score, and RDKit filters such as PAINS,^47^ Brenk,^48^ and NIH^38,39^ filters. After filtering, 3061 molecules remained. Next, we checked the generated molecules with PoseBusters, refining the set further to 2716 molecules, of which 1139 were unique.

We then evaluated these molecules based on their minimized Vina docking scores. Among the 1139 unique molecules, 55.5% exhibited the same or greater interactions as the developed compound by Yan et al. (measured with ODDT) with an overall 98% showing more interactions. In addition, 82% of these molecules had minimized Vina scores better than the developed compound (AtC-45) and 10% had better minimized Vina scores than the biapenem product. We also examined the structural similarity of the generated molecules to the developed compound, despite the reference points not being fully aligned with it, and found that 125 ligands had SC_RDKit_ scores greater than 0.5. This demonstrates that MolSnapper successfully generated ligands similar to the developed compound, with comparable interactions. In Figure 4, the best-scoring molecules based on SC_RDKit_ are presented, along with a comparison of interactions between the developed compound and MolSnapper-generated molecules. Three of the four molecules had better docking scores than AtC-45, and the Vina score of the molecule with the best SC_RDKit_ score exceeded the biapenem product.

In contrast, following the same pipeline using SILVR resulted in 1376 molecules that passed the QED, SA, and RDKit filtering. After applying PoseBusters, 920 molecules remained, with 752 unique, but none had SC_RDKit_ scores greater than 0.5, and none had Vina scores better than the developed compound.

#### Case Study 2: Hit Optimization with Scaffold Constraints

The COVID-19 pandemic, caused by SARS-CoV-2, has led to millions of deaths and continues to challenge global health. Despite the rapid development of vaccines, there is still an urgent need for effective oral antiviral drugs to combat the virus and its variants.

Key interactions observed in the Moonshot compounds^49,50^ led to the identification of several hits selected using docking-based virtual screening.^51^ Unoh et al. optimized one of these initial hits (Hit Compound 1, PDB ID: 7VTH) using a traditional structure-based drug design strategy to yield compound S-217622, the first oral noncovalent, nonpeptidic SARS-CoV-2 3CL protease (3CL_pro_) inhibitor clinical candidate. The design strategy employed to advance the initial hit focused on optimizing the R-group around a core scaffold to enhance binding affinity and specificity by improving the existing hydrogen bonds and other intermolecular interactions.

**Figure 5:**
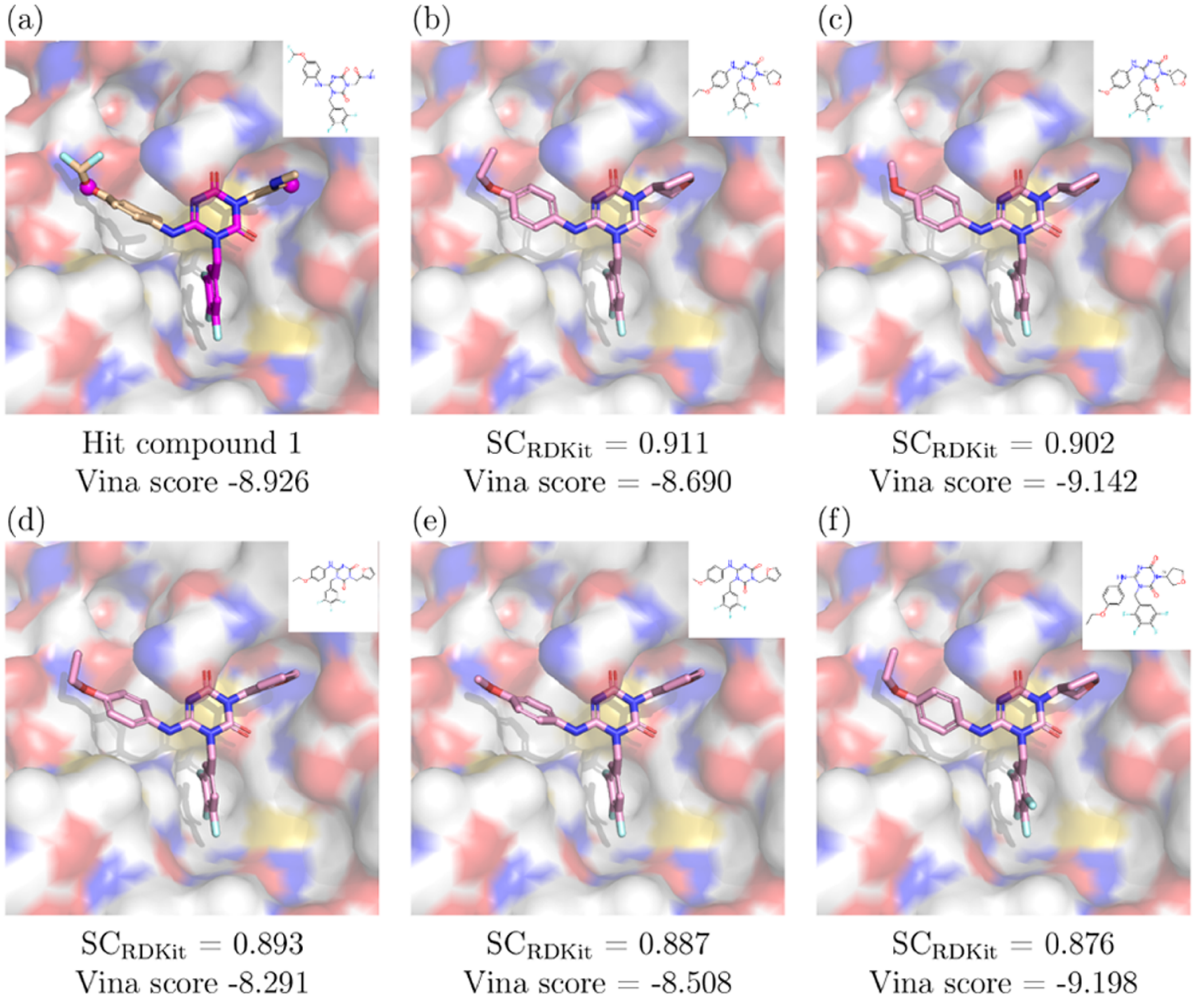
Case Study 2: COVID-19 3CL^pro^ hit-to-lead. (a) Hit compound 1 from Unoh et al.^51^ (PDB ID: 7VTH), with the scaffold and reference pharmacophores for MolSnapper (magenta). (b–f) The top 5 molecules generated by MolSnapper by SC_RDKit_ score. Minimized Vina scores are shown for all complexes.

We adopted a similar approach to Unoh et al.^51^ and chose to constrain the same core scaffold of Hit Compound 1 while optimizing two hydrogen bonding interactions exhibited in the crystal structure of Hit Compound 1 in complex with 3CL^pro^. Both the constraints and Hit Compound 1 are shown in Figure 5a. Using MolSnapper, we generated 5000 molecules and applied a series of filters, including QED, SA score, NIH, and PAINS, which reduced the set to 2413 molecules. From these, 1234 passed PoseBusters, with 1077 being unique. 49% of the unique molecules recapitulated the exact interactions observed in the reference ligand (to the same residue), while 77% demonstrated equal or additional interactions (not necessarily to the same residues). In addition, 8% of these molecules had minimized Vina docking scores better than Hit Compound 1.

In Figure 5, we present the top 5 generated molecules that most closely resemble the reference structure based on SC_RDKit_ score. All of these molecules share the same key interactions as the reference compound, and two have minimized Vina scores that exceed Hit Compound 1.

This case study illustrates the flexibility of MolSnapper, demonstrating that it can be effectively used not only for generating whole molecules but also for scaffold growth, allowing for the refinement and enhancement of existing scaffolds to achieve desired interactions.

### Ablation Studies

We compared three variations of initialization strategies and the treatment of reference points on the CrossDocked data set.

1. Random Initialization around Pharmacophore Fixed Point with Fixed Reference Positions: The generated molecule’s initial positions are random noise around the pharmacophore fixed points and the reference positions are fixed during the diffusion reverse process. This setting corresponds to the choices used in MolSnapper.
2. Random Initialization with Fixed Reference Positions: The generated molecule’s initial positions are random noise and the reference positions remain fixed throughout the generation process as in the previous scenario.
3. Random Initialization around Pharmacophore Fixed Point with Noised Reference Points: The generated molecule’s initial positions are random noise around the pharmacophore fixed points and the reference points are noised based on a predefined noise scaling schedule during the diffusion reverse process.

Table 3 summarizes our ablation study results, focusing on the impact of noise in molecular generation using GNNs. Optimal SC_RDKit_ scores were achieved with initialization 1, random initialization around fixed pharmacophore points, but without adding noise to reference points. This suggests the importance of stable reference points in maintaining key ligand-target interactions during the generation process.

**Table 3:** Ablation Studies Comparing Three Distinct Strategies for Initializing and Conditioning Molecular Generation in the CrossDocked Data Set

	Pass	SC_RDKit_ ↑	SA ↑		Interaction Sim. ↑	
Method	Rate↑	Top 1	Top 1	Top 3	Top 1	Top 3
MolSnapper	**58%**	**0.714 ± 0.116**	**0.642 ± 0.182**	0.722 ± 0.162	**0.746 ± 0.246**	0.824 ± 0.204
(2) Random Init.	**58%**	0.709 ± 0.108	0.638 ± 0.1180	**0.735 ± 0.157**	0.740 ± 0.245	**0.842 ± 0.197**
(3) Noised ref.	54%	0.705 ± 0.102	0.622 ± 0.208	0.715 ± 0.164	0.736 ± 0.243	0.809 ± 0.220

### Pose Consistency Upon Redocking

Redocking is commonly used to produce new poses for molecules generated by methods that produce binding site conditioned poses.^26^ While this is common practice and potentially useful in cases where the generated molecule is promising but the generated pose is poor, the redocking step does not preserve the information from the pose generated by the conditioning method. Thus, assessment using redocked poses does not allow us to assess the quality of the generated poses. Recently, PoseCheck^26^ demonstrated both that the generated poses change significantly and that the redocked poses can mask nonphysical features of the generated poses, for instance, steric clashes, hydrogen placement issues, and high strain energies. Thus, our evaluation of MolSnapper did not involve redocking, although we did perform energy minimization, as is typically recommended even for experimentally determined structures.

However, despite achieving reasonable docking scores both with and without minimization (Figures S3, S7, and S8), it is informative to confirm that the generated poses are similar to a low-energy pose generated through redocking. We docked all 1139 unique molecules that passed the filters in Case Study 1 and all 1077 unique molecules that passed the filters in Case Study 2. We found that around 95% and 98%, respectively, of all generated molecules had a docked pose within 2 Å of the generated pose (Figure S13). To ensure these findings were more broadly applicable, we additionally checked the RMSD upon redocking for the top molecule for each target according to SC_RDKit_ for the CrossDocked data set. The MolSnapper-generated molecules had an average RMSD of 2.31 Å (52% ≤ 2 Å) while the DiffSBDD-generated molecules had an average RMSD of 1.82 Å (70% ≤ 2 Å). This shows that the majority of generated molecules for both MolSnapper and DiffSBDD have a low-energy pose within 2 Å per the Vina scoring function.

## Conclusions

We present MolSnapper, a novel method for conditioning diffusion models to incorporate 3D pharmacophoric constraints, offering a tool for molecular generation with controlled design processes. MolSnapper readily enables the utilization of prior knowledge to improve molecule design.

In our experiments on multiple data sets, we demonstrated that MolSnapper outperforms competing methods, SILVR and DiffSBDD, producing up to 3 times more molecules that pass PoseBusters checks. It also offers up to a 20% improvement in the shape and color similarity to the reference ligands, leading to a 30% better retrieval of initial hits. Additionally, through two case studies, we highlighted the applicability of MolSnapper to both early stage drug discovery (Case Study 1), performing de novo design with only target pharmacophores, and lead optimization (Case Study 2), generating molecules with constrained scaffolds that also make specific additional intermolecular interactions.

As is the case with many generative models, some of the molecules generated by MolSnapper might be challenging to synthesize. Furthermore, none of the methods considered in this manuscript include an explicit mechanism to assess or enforce the generation of molecules in specific tautomeric or protomeric forms. While reaction-based generative models that can propose molecules together with a synthetic route exist (e.g. Bradshaw et al.,^52^ such approaches remain nascent and currently lack flexibility. We demonstrated that MolSnapper generated molecules with improved synthetic accessibility scores compared to previous pocket-conditioned generative approaches.

We assessed the generated molecules and their poses in several ways, including physical validity checks, comparisons to ground-truth ligands, the ability to generate molecules with desired interactions, and molecular docking. While these metrics assess several facets of the generated poses, new methods and metrics are needed for rigorously assessing the capabilities of SBDD-based generative methods that generate 3D poses.

We primarily used known binders as sources from which to infer pharmacophores and other constraints in our experiments. However, this is not a requirement, and other approaches can be used in the absence of a known binder. In such cases, candidate pharmacophores and other constraints could be determined by other computational approaches, such as fragment hotspots^53^ as employed by STRIFE,^24^ or specified by medicinal chemists.

Our work bridges a crucial gap as it enables the use of models trained on large data sets of molecules to be conditioned for use in pocket binding without the requirement for training specifically on protein–ligand complexes. Our approach generates ligands in a controlled and effective generative process, integrating 3D structural information and expert knowledge.

## Data Availability

The code developed for this study is available at https://github.com/oxpig/MolSnapper. The data sets used, including CrossDocked^31^ and Binding MOAD,^32^ are open-source, and the filtered complexes prepared for use with our tool can be accessed at https://github.com/oxpig/MolSnapper/tree/master/data. Detailed instructions for downloading and using these resources are also provided at https://github.com/oxpig/MolSnapper. All data are provided in a machine-readable format to ensure reproducibility and accessibility, following the guidelines on data sharing and reproducibility.

## Supporting Information Available

Lists of the filtered ground truth ligands used for evaluation from the CrossDocked test data set and the Binding MOAD test data set; a table (Table S1) showing the comparison on the CrossDocked data set of MolDiff (without conditioning, docked using the Vina docking method), DiffSBDD (trained on CrossDocked), and our method, MolSnapper; detailed implementation information, including specific filters applied for the case studies; and additional figures; distributions of properties of the generated molecules in Figures S1, S2, S4, S5, S6, S9, S11; further examples of generated molecules in Figures S10, S12; docking scores in Figures S3, S7, and S8; RMSD upon redocking in Figure S13 (PDF)
Simplified Molecular Input Line Entry System (SMILES) for compounds in the manuscript (XLSX)
