# Supplementary material for: MolSnapper: Conditioning Diffusion for Structure-Based Drug Design
Source: J Chem Inf Model. 2025 Apr 18;65(9):4263–73. doi: 10.1021/acs.jcim.4c02008 (PMC12076506; doi:10.1021/acs.jcim.4c02008)
Supplement: Supplementary file 1 — ci4c02008_si_001.pdf [file ci4c02008_si_001.pdf]

Supporting Information
MolSnapper: Conditioning Diffusion for Structure Based Drug Design

Yael Ziv,[†] Fergus Imrie,[†] Brian Marsden,[‡] and Charlotte M. Deane[*,†]

†*Department of Statistics, University of Oxford, St Giles', OX1 3LB, Oxford, United Kingdom*

‡*Nuffield Department of Medicine, University of Oxford, Old Road, OX3 7BN, Oxford, United Kingdom*

E-mail: deane@stats.ox.ac.uk

S1 Filtered Ground Truth Ligands from CrossDocked test dataset

1. 2z3h_A_rec_1wn6_bst_lig_tt_docked_3
2. 4aaw_A_rec_4ac3_r83_lig_tt_min_0
3. 4yhj_A_rec_4yhj_an2_lig_tt_min_0
4. 14gs_A_rec_20gs_cbd_lig_tt_min_0
5. 4rn0_B_rec_4rn1_l8g_lig_tt_min_0
6. 1fmc_B_rec_1fmc_cho_lig_tt_docked_1
7. 3daf_A_rec_3daf_feg_lig_tt_docked_0
8. 5w2g_A_rec_5w2i_adp_lig_tt_min_0
9. 3dzh_A_rec_3u4i_cvr_lig_tt_docked_0
10. 3g51_A_rec_3g51_anp_lig_tt_min_0
11. 2jjg_A_rec_2jjg_plp_lig_tt_min_0
12. 2rhy_A_rec_2rhy_mlz_lig_tt_min_0
13. 2pqw_A_rec_2rhy_mlz_lig_tt_min_0
14. 5bur_A_rec_5x8f_amp_lig_tt_docked_7
15. 3gs6_A_rec_2oxn_oan_lig_tt_docked_4
16. 1r1h_A_rec_1r1h_bir_lig_tt_docked_1
17. 1dxo_C_rec_1gg5_e09_lig_tt_min_0

18. 1gg5_A_rec_1kbo_340_lig_tt_min_0
19. 5b08_A_rec_5b09_4mx_lig_tt_min_0
20. 2azy_A_rec_2azy_chd_lig_tt_docked_0
21. 5i0b_A_rec_5vef_m77_lig_tt_min_0
22. 1phk_A_rec_1phk_atp_lig_tt_min_0
23. 4keu_A_rec_4ket_pg4_lig_tt_min_0
24. 1djy_A_rec_1djz_ip2_lig_tt_min_0
25. 5l1v_A_rec_5l1v_7pf_lig_tt_docked_0
26. 2rma_A_rec_3rdd_ea4_lig_tt_docked_0
27. 4p6p_A_rec_4p77_5rp_lig_tt_docked_0
28. 3u5y_B_rec_3u57_dh8_lig_tt_min_0
29. 4f1m_A_rec_4f1m_acp_lig_tt_min_0
30. 4tqr_A_rec_2xca_doc_lig_tt_min_0
31. 4lfu_A_rec_4y13_480_lig_tt_min_0
32. 3jyh_A_rec_3n0t_opy_lig_tt_min_0
33. 4iwq_A_rec_4jlc_su6_lig_tt_min_0
34. 1l3l_A_rec_1l3l_lae_lig_tt_min_0
35. 1e8h_A_rec_1e8h_adp_lig_tt_min_0
36. 2e24_A_rec_1j0n_ceg_lig_tt_docked_14
37. 2hcj_B_rec_2hcj_gdp_lig_tt_docked_0

38. 3kc1_A_rec_3kc1_2t6_lig_tt_min_0

39. 4ja8_B_rec_4ja8_1k9_lig_tt_docked_0

40. 4iiy_A_rec_3tle_gsu_lig_tt_docked_12

41. 3v4t_A_rec_4e7f_udp_lig_tt_min_0

42. 3tym_A_rec_3n5v_xfh_lig_tt_min_0

43. 4d7o_A_rec_3n5z_xfm_lig_tt_min_0

44. 4kcq_A_rec_4cwv_hw8_lig_tt_min_0

45. 1umd_B_rec_1umb_tdp_lig_tt_docked_1

46. 4pxz_A_rec_4pxz_6ad_lig_tt_min_0

47. 2cy0_A_rec_2d5c_skm_lig_tt_min_0

48. 3w83_B_rec_2e6d_fum_lig_tt_min_0

49. 2e6d_A_rec_2e6d_fum_lig_tt_min_0

50. 4rv4_A_rec_4rv4_prp_lig_tt_docked_2

51. 5d7n_D_rec_4jt9_1ns_lig_tt_min_0

52. 4tos_A_rec_4tos_355_lig_tt_min_0

53. 5aeh_A_rec_5aeh_8ir_lig_tt_docked_0

54. 4rlu_A_rec_4rlu_hcc_lig_tt_min_0

55. 4xli_B_rec_4xli_1n1_lig_tt_min_0

56. 3l3n_A_rec_2iux_nxa_lig_tt_docked_2

57. 5tjn_A_rec_1zj1_nlc_lig_tt_docked_4

58. 5liu_X_rec_4gq0_qap_lig_tt_min_0
59. 3o96_A_rec_3o96_iqo_lig_tt_docked_2
60. 4qlk_A_rec_4qlk_ctt_lig_tt_docked_0
61. 3hy9_B_rec_3hyg_099_lig_tt_min_0
62. 4bel_A_rec_2ewy_dbo_lig_tt_min_0
63. 3nfb_A_rec_3nfb_oae_lig_tt_docked_2
64. 4m7t_A_rec_4m7t_sam_lig_tt_min_0
65. 3u9f_C_rec_3u9f_clm_lig_tt_min_0
66. 2f2c_B_rec_1xo2_fse_lig_tt_min_0
67. 3chc_B_rec_3ch9_xrg_lig_tt_min_0
68. 4z2g_A_rec_4z2g_m6v_lig_tt_docked_16
69. 3af2_A_rec_3af4_gcp_lig_tt_min_0
70. 1jn2_P_rec_1val_png_lig_tt_docked_11
71. 3li4_A_rec_2gvv_di9_lig_tt_min_0
72. 4azf_A_rec_5lxc_7aa_lig_tt_min_0
73. 2pc8_A_rec_1eqc_cts_lig_tt_docked_6

S2 Filtered Ground Truth Ligands from Binding MOAD test dataset

1. 7AKI-bio1_RJQ_A_202

2. 2FKY-bio2_N2T_B_605
3. 3EKU-bio1_CY9_A_903
4. 1RDW-bio1_LAR_X_391
5. 5NCP-bio1_EG5_A_201
6. 7AKI-bio1_RJQ_A_203
7. 5NEG-bio1_8VK_B_201
8. 5NCG-bio1_8TB_A_202
9. 6RCJ-bio1_K0H_A_201
10. 2FL6-bio2_N5T_B_605
11. 3F7H-bio2_419_B_1
12. 3F7I-bio2_G13_B_1
13. 2I3I-bio1_618_A_501
14. 3GTA-bio2_851_B_1
15. 2Q0U-bio1_LAB_A_401
16. 2FKY-bio1_N2T_A_604
17. 2I3I-bio2_618_B_501
18. 3EKS-bio1_CY9_A_903
19. 5NCG-bio2_8TB_B_202
20. 3CJO-bio1_K30_A_1
21. 5XDU-bio1_ZI6_A_403

22. 1ESV-bio1_LAR_A_401

23. 4PA0-bio2_2OW_B_1101

24. 3GT9-bio2_516_B_1

25. 3GTA-bio1_851_A_1

26. 5NCG-bio2_8TB_B_201

27. 5NDU-bio2_8V2_B_201

28. 2A5X-bio1_LAR_A_379

29. 2Q2Y-bio1_MKR_A_604

30. 5NCG-bio1_8TB_A_201

31. 3GT9-bio1_516_A_1

32. 1SQK-bio1_LAR_A_378

33. 6RCF-bio1_K0K_A_201

34. 3F7H-bio1_419_A_1

35. 5NCF-bio2_8T5_B_204

36. 4PA0-bio1_2OW_A_1101

37. 5ZZB-bio2_LAB_D_401

38. 2FL2-bio2_N4T_B_605

39. 5NDU-bio1_8V2_A_201

40. 2Q2Y-bio2_MKR_B_605

41. 5NEG-bio1_8VK_A_201

42. 3F7I-bio1_G13_A_1

43. 3MN5-bio1_LAB_A_376

Table S1: Comparison on the CrossDocked dataset of MolDiff (without conditioning, which was docked with the Vina docking method), DiffSBDD (trained on CrossDocked), and our method, MolSnapper. Arrows next to metrics indicate superiority (up: larger is better, down: smaller is better). The best results are highlighted in bold. 'Pass Rate' refers to the percentage of ligands passing the PoseBusters test. 'SC_{RDKit}' similarity to the ground truth ligand. 'SA' stands for Synthetic Accessibility. 'Same ODDT Interaction' assesses the similarity in hydrogen bonding interactions compared to a reference.

	MolDiff	DiffSBDD (CrossDocked)	MolSnapper	MolSnapper redocked
Pass Rate ↑	**92% ± 15%**	47% ± 19%	58% ± 22%	61% ± 21%
SC_{RDKit} Top 1 ↑	0.421 ± 0.109	0.628 ± 0.141	**0.714 ± 0.116**	**0.714 ± 0.116**
SA Top 1 ↑	**0.86 ± 0.096**	0.638 ± 0.118	0.642 ± 0.182	0.642 ± 0.182
SA Top 5 ↑	**0.916 ± 0.060**	0.695 ± 0.094	0.722 ± 0.162	0.722 ± 0.162
Interaction Sim. Top 1 ↑	0.284 ± 0.217	**0.783 ± 0.219**	0.746 ± 0.246	0.527 ± 0.311
Interaction Sim. Top 5 ↑	0.496 ± 0.262	**0.874 ± 0.160**	0.824 ± 0.204	0.716 ± 0.236

S3 Implementation details

In our experiments on CrossDocked and Binding MOAD, we enforced that nitrogen (N) is selected for donor atoms, and oxygen (O) is chosen for acceptor atoms.

S3.1 Docking protocols

We used AutoDock Vina[1] to dock and score molecules. We docked molecules that were not generated with a pose conditioned on the binding site and initially scored molecules with binding site conditional poses without further structural refinement or minimization. As previously stated, since scoring functions are sensitive to the exact coordinates and can assign experimental structures poor scores without energy minimization, we ultimately primarily computed energy-minimized scores for generated molecules with binding site conditional poses.[2] However, comparisons between unminimized and minimized scores are shown in Figures S3, S7, and S8.

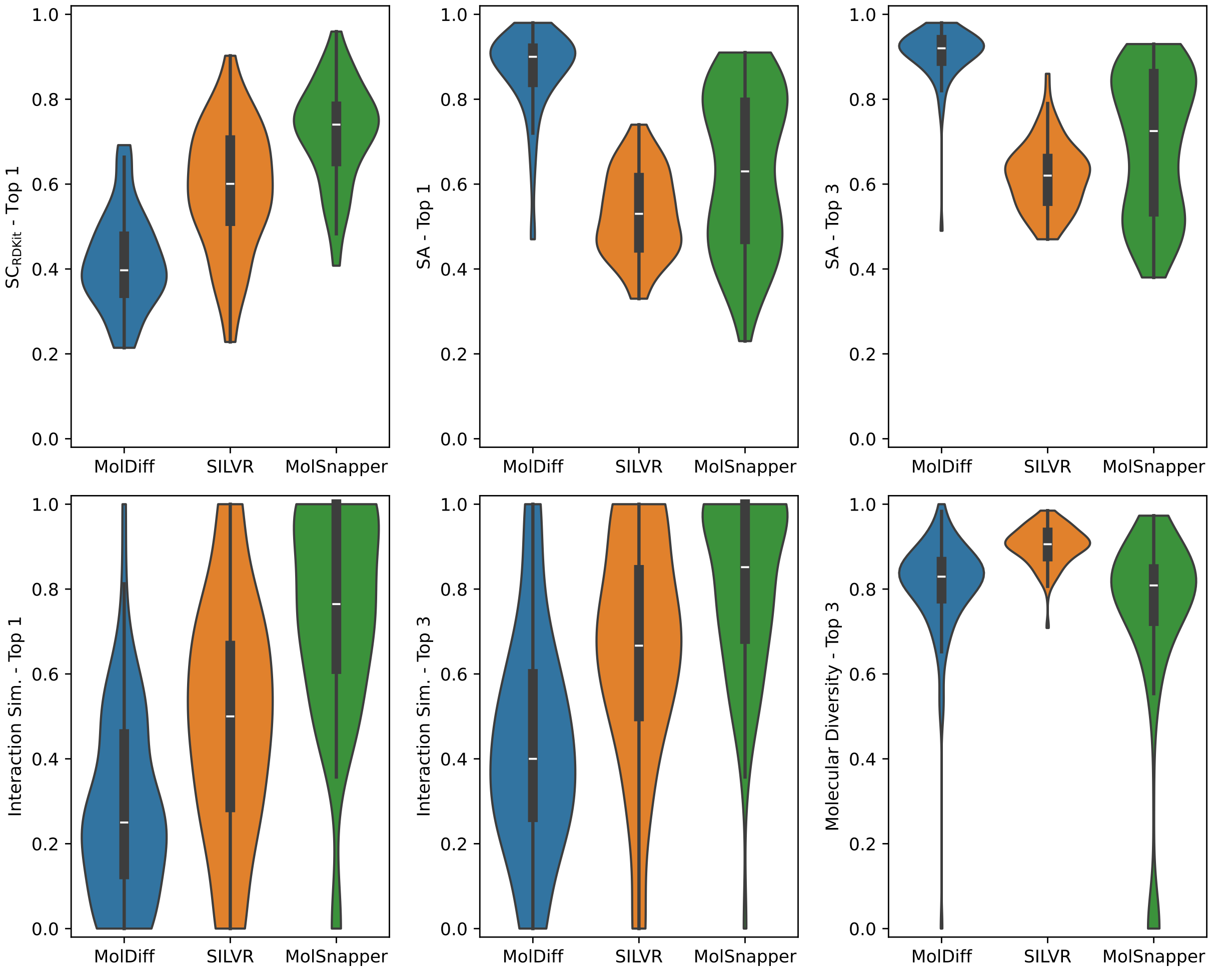

Figure S1: Comparison on the CrossDocked dataset of MolDiff (without conditioning), SILVR, and our method, MolSnapper. Metrics are defined in Methods - Experimental Setup - Evaluation.

Specifically, to compute Vina Scores, we used the implementation from DecompDiff[3] ('https://github.com/bytedance/DecompDiff/blob/main/scripts/evaluate_mol_from_meta_full.py'). We used the default settings provided in their implementation, ensuring consistency with their methodology. Their protocol first prepares the protein structure using AutoDock tools and the ligand using the preparation pipeline provided in the meeko Python package. Then, AutoDock Vina is used to compute docking scores for generated molecules using the AutoDock Vina Python API.

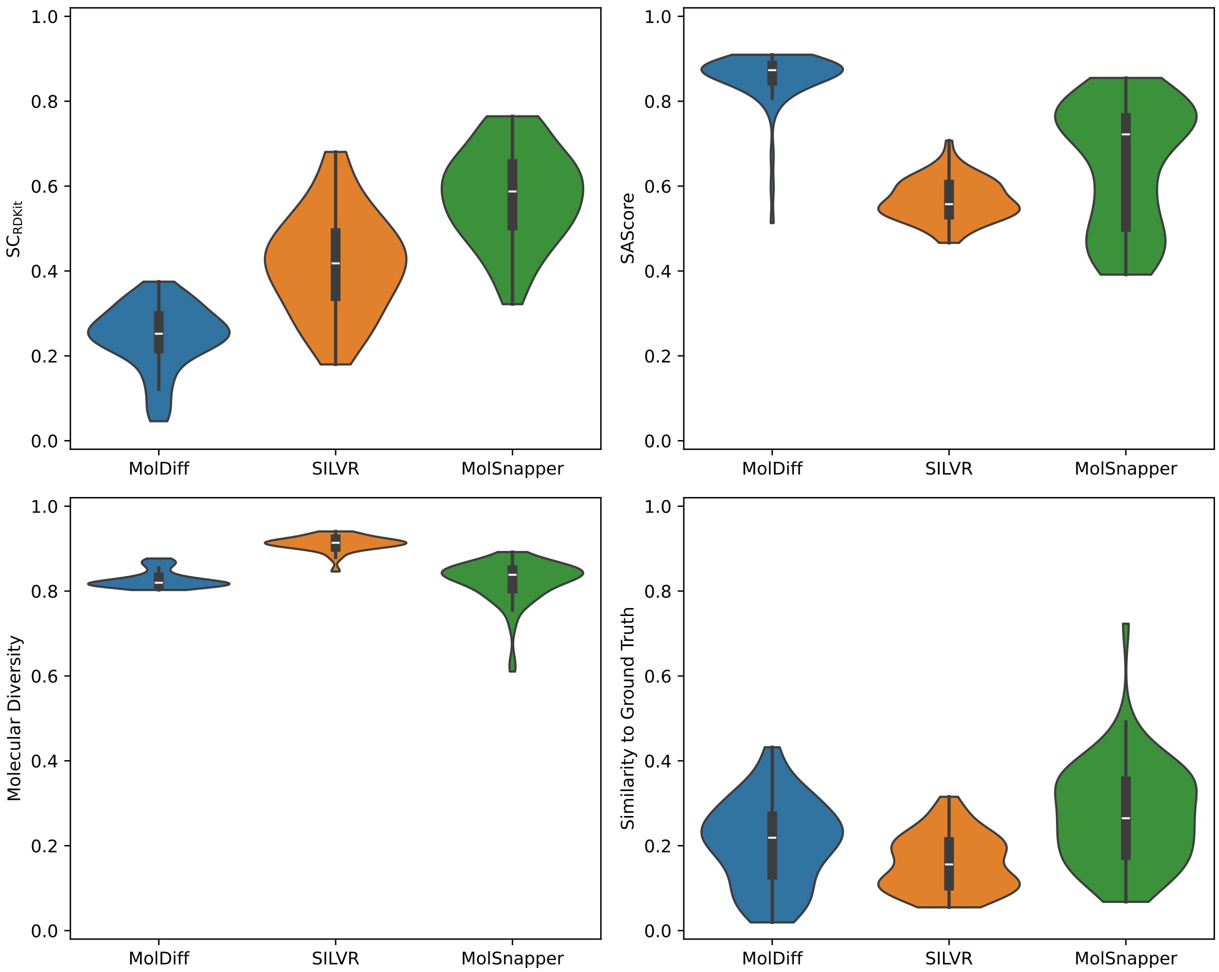

Figure S2: Comparison on the CrossDocked dataset of MolDiff (without conditioning), SILVR, and our method, MolSnapper. Metrics calculated using all molecules that pass PoseBusters validity checks. Metrics are defined in Methods - Experimental Setup - Evaluation.

S3.2 Case study 1

The PAINS filter was applied to exclude molecules likely to cause nonspecific binding and false positives, while the Brenk filter was used to remove substructures associated with poor pharmacokinetics or toxicity. Additionally, the NIH filter provided an extra layer of safety screening. The thresholds used were QED ≥ 0.25 and SA score≥ 0.5.

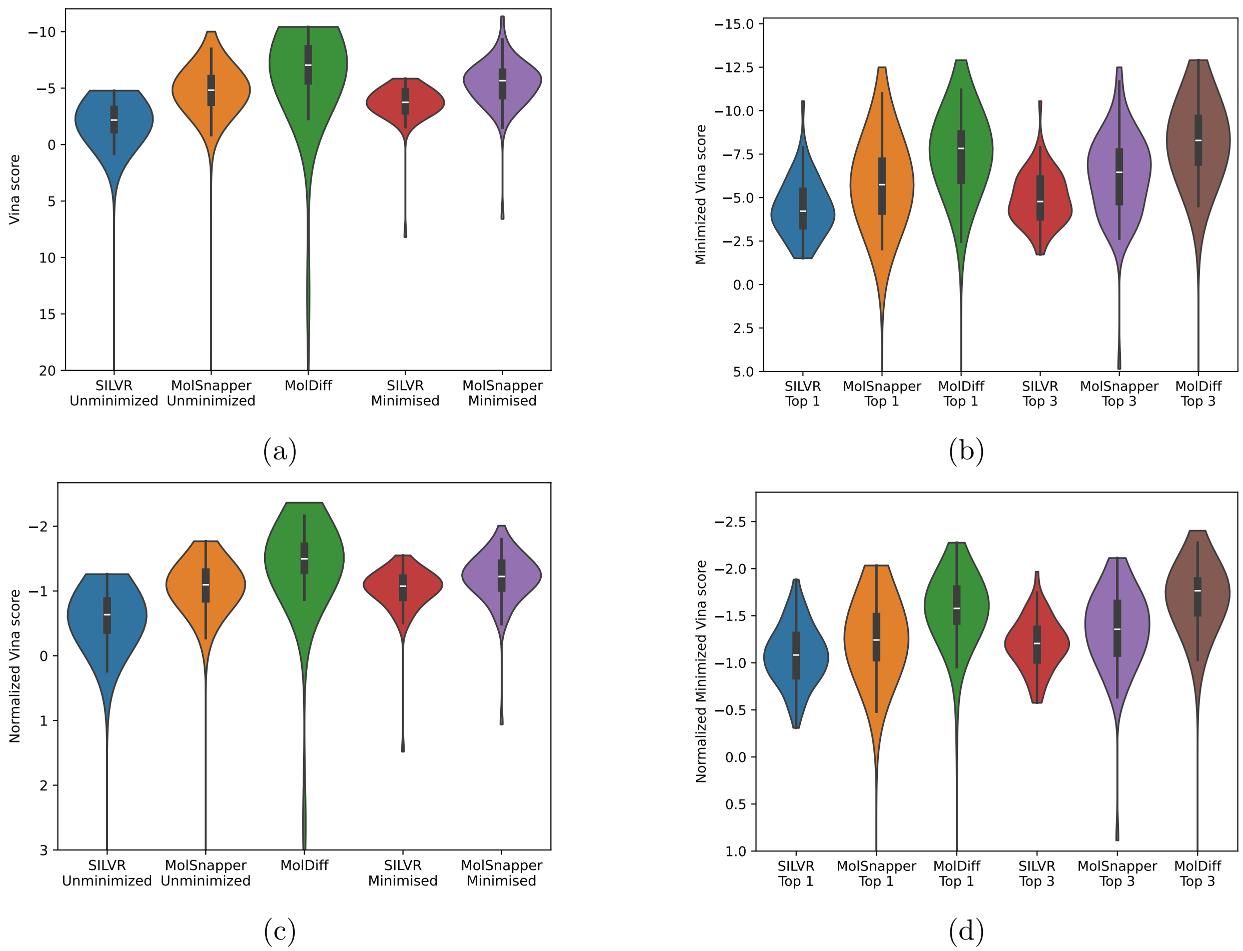

Figure S3: Comparison of docking scores on CrossDocked dataset of MolDiff (without conditioning), SILVR, and our method, MolSnapper. (a) Comparison of unminimized and minimized Vina scores across all molecules that passed PoseBusters validity checks. (b) Comparison of minimized Vina scores for the top 1 and top 3 molecules by SC_{RDKit} scores. (c) and (d) mirror (a) and (b), respectively, but for normalized Vina scores.

S3.3 Case study 2

In this case, only the PAINS and NIH filters were applied, as the reference molecule did not pass the Brenk filter. The same thresholds were used: QED ≥ 0.25 and SA score≥ 0.5.

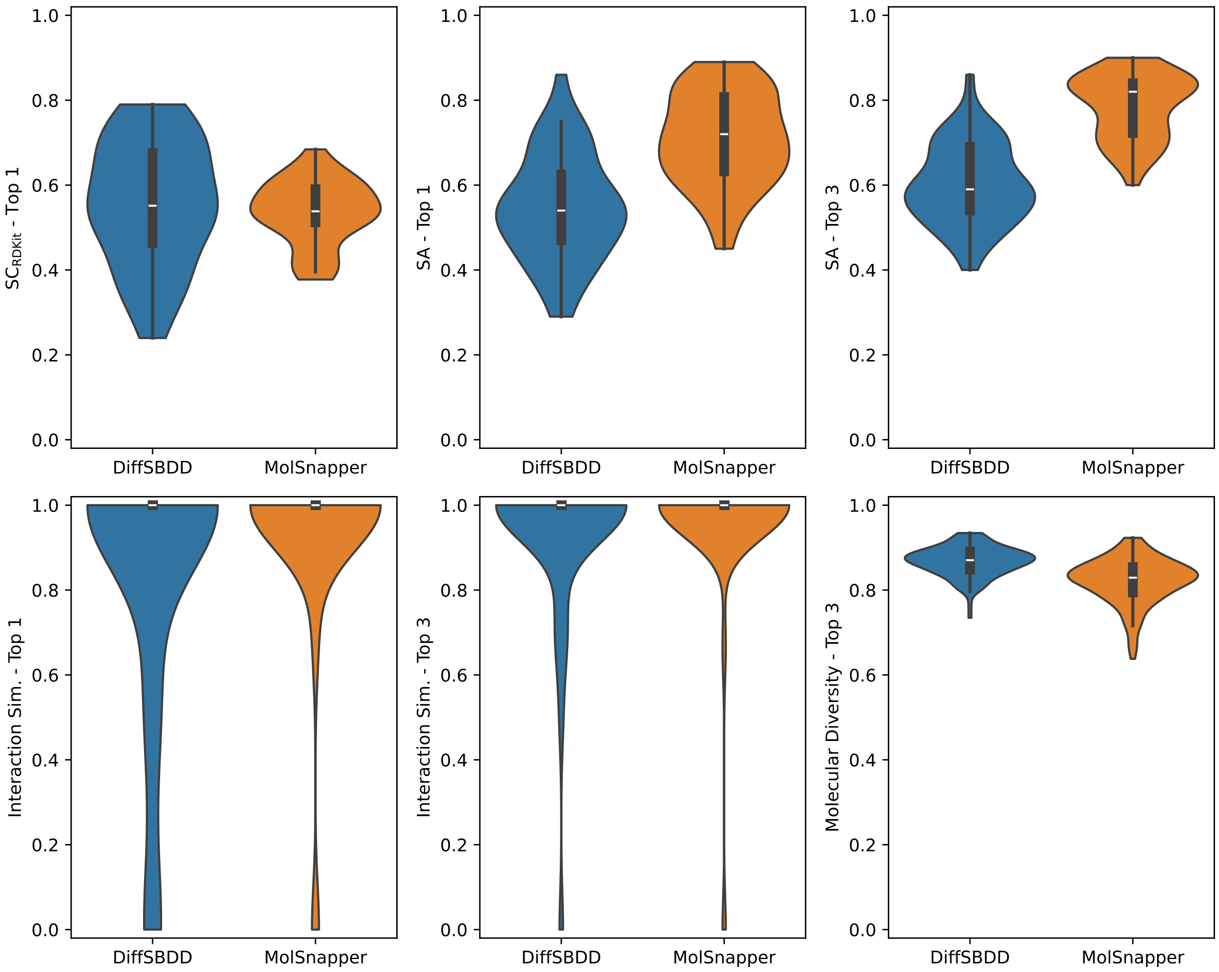

Figure S4: Comparison on Binding MOAD of DiffSBDD (trained on Binding MOAD) and our method, MolSnapper. Metrics are defined in Methods - Experimental Setup - Evaluation.

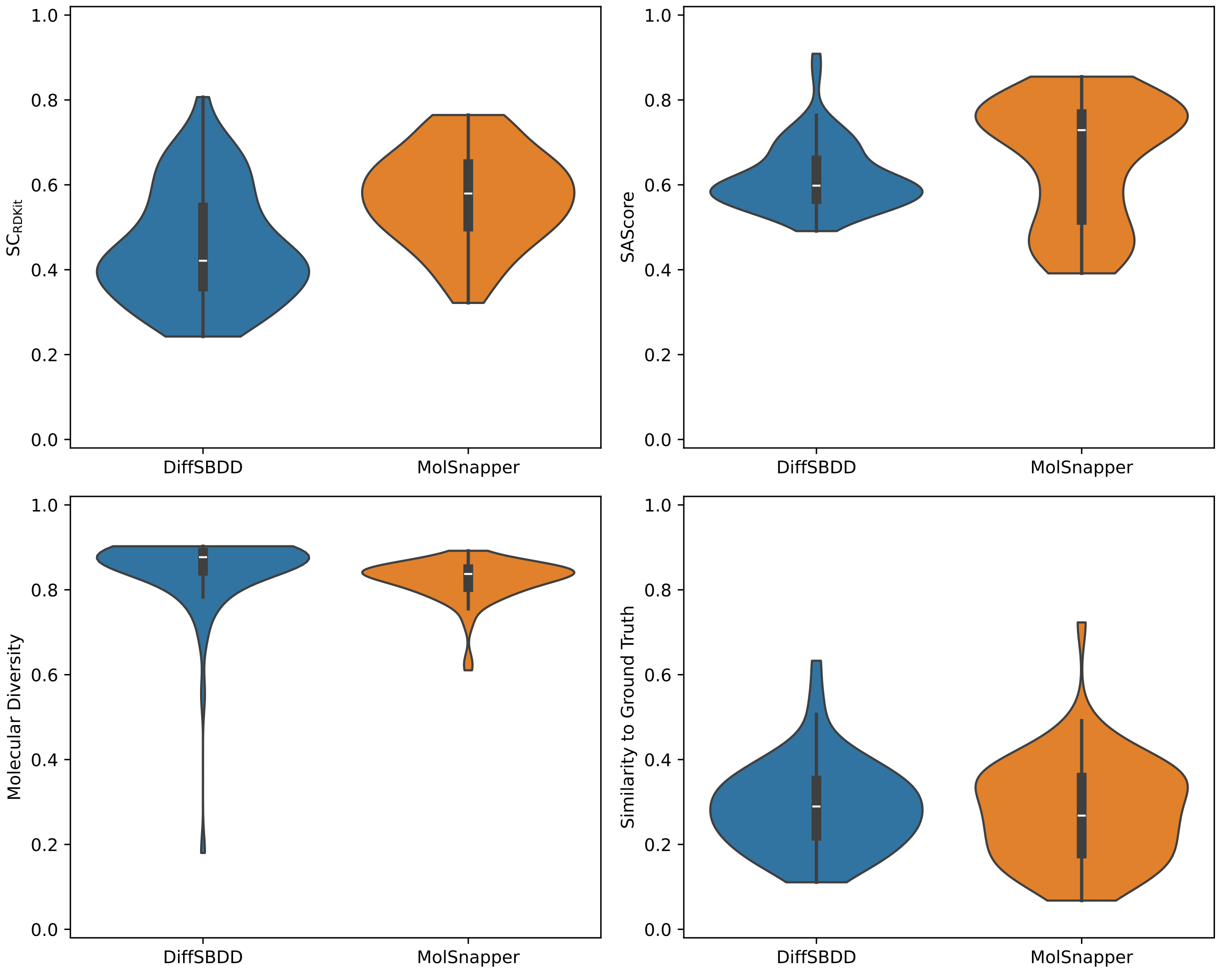

Figure S5: Comparison on the CrossDocked dataset of DiffSBDD (trained on CrossDocked) and our method, MolSnapper. Metrics calculated using all molecules that pass PoseBusters validity checks. Metrics are defined in Methods - Experimental Setup - Evaluation.

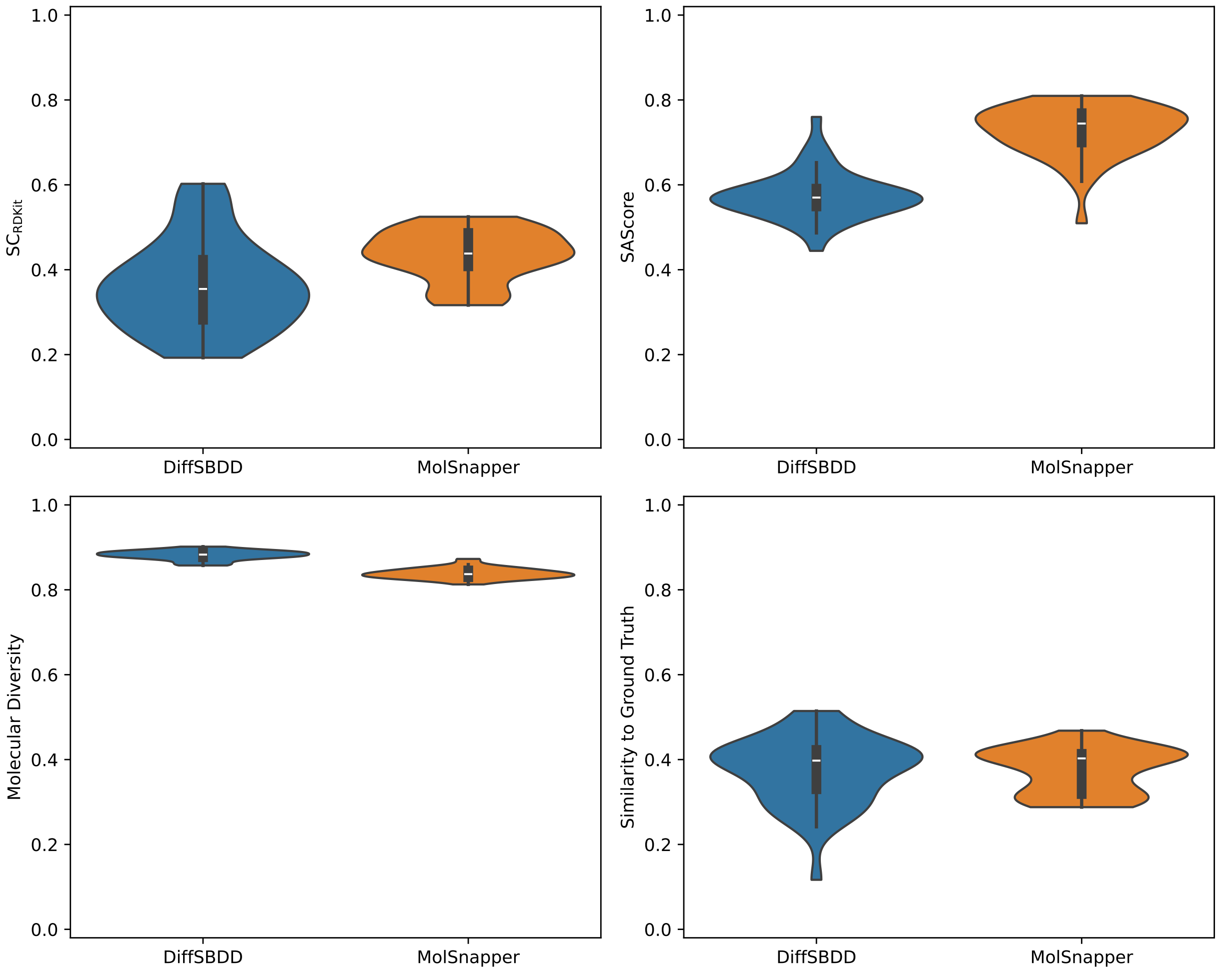

Figure S6: Comparison on Binding MOAD of DiffSBDD (trained on Binding MOAD) and our method, MolSnapper. Metrics calculated using all molecules that pass PoseBusters validity checks. Metrics are defined in Methods - Experimental Setup - Evaluation.

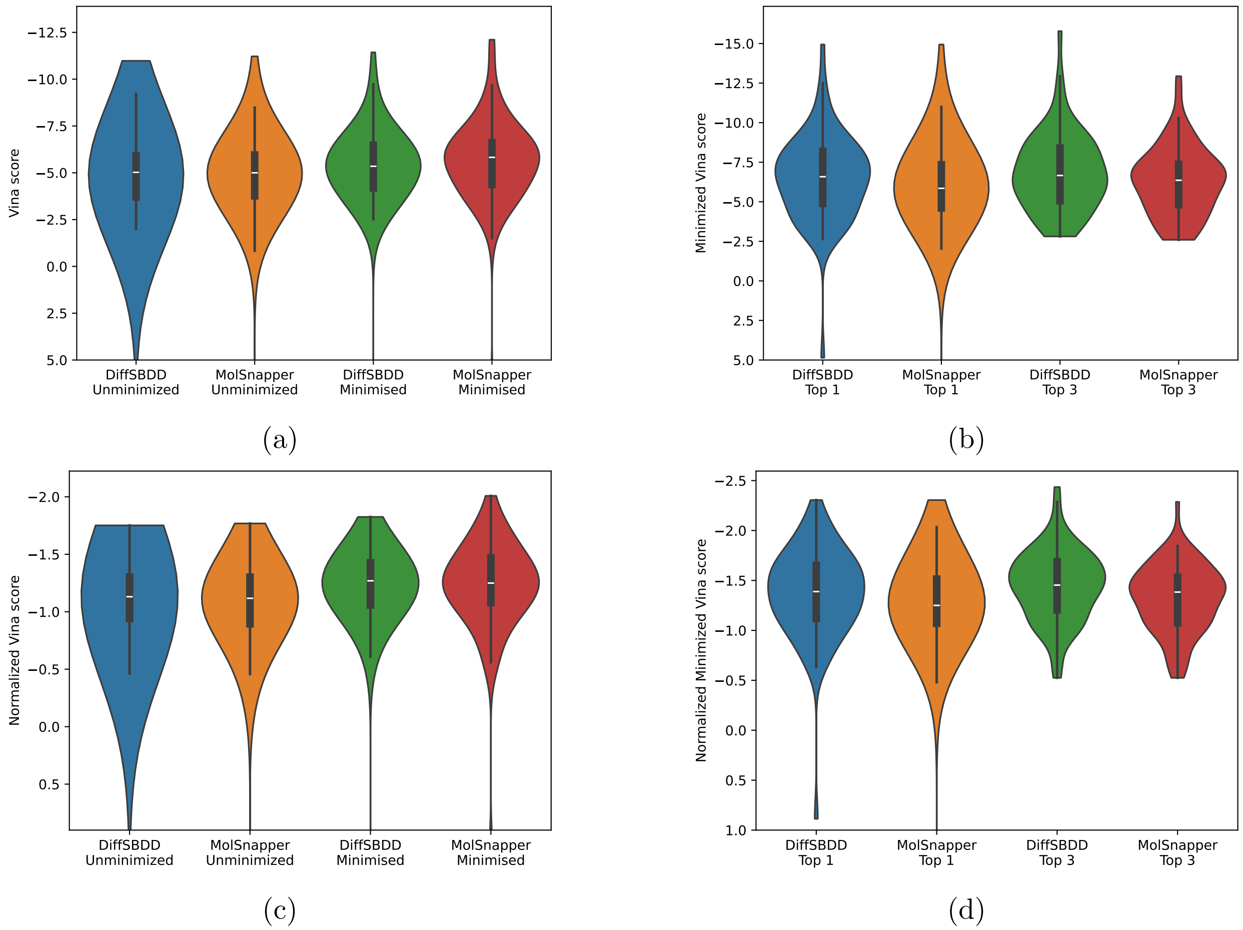

Figure S7: Comparison of docking scores on the CrossDocked dataset of DiffSBDD (trained on CrossDocked) and our method, MolSnapper. (a) Comparison of unminimized and minimized Vina scores across all molecules that passed PoseBusters validity checks. (b) Comparison of minimized Vina scores for the top 1 and top 3 molecules by SC_{RDKit} scores. (c) and (d) mirror (a) and (b), respectively, but for normalized Vina scores.

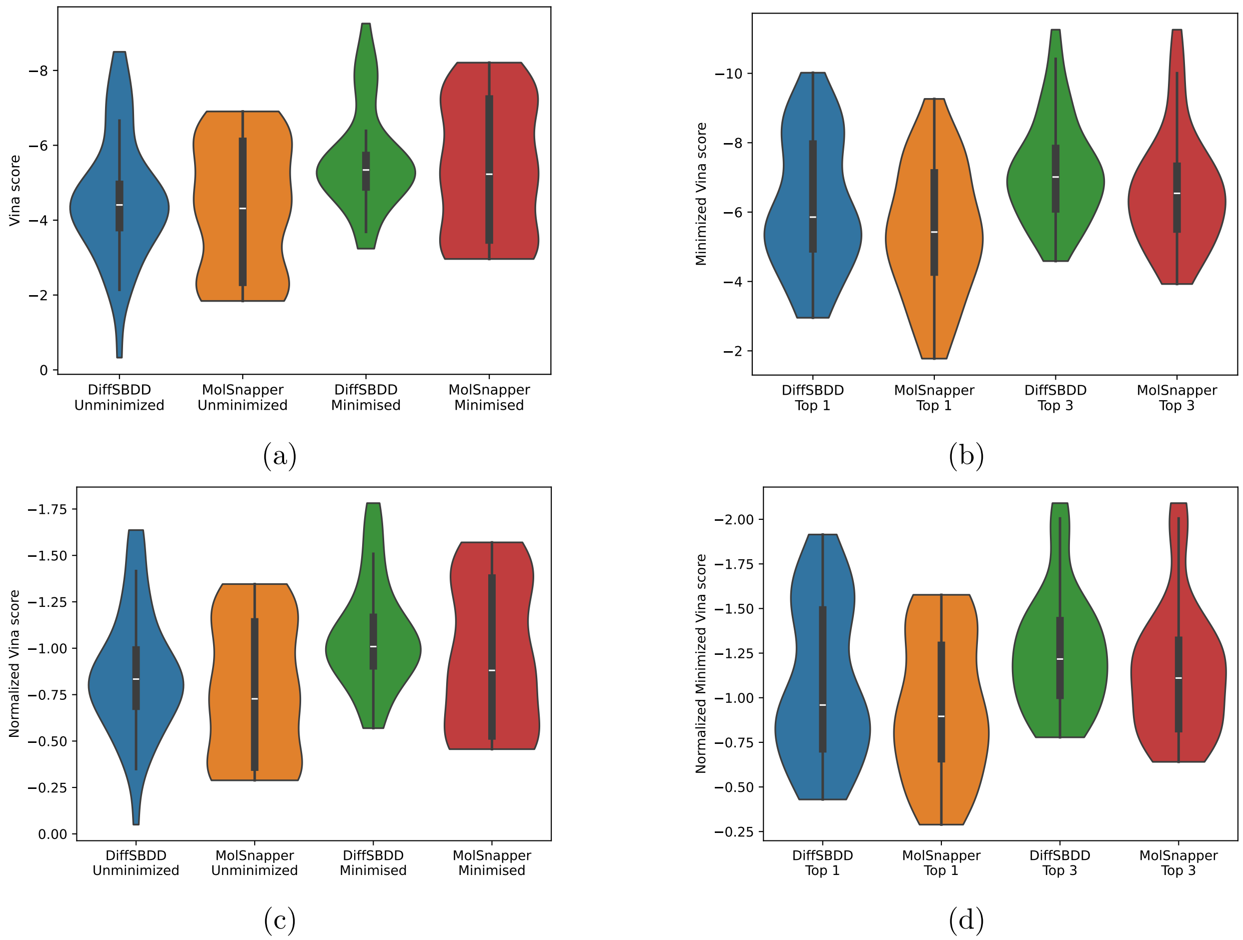

Figure S8: Comparison of docking scores on BindingMOAD of DiffSBDD (trained on Binding MOAD) and our method, MolSnapper. (a) Comparison of unminimized and minimized Vina scores across all molecules that passed PoseBusters validity checks. (b) Comparison of minimized Vina scores for the top 1 and top 3 molecules by SC_{RDKit} scores. (c) and (d) mirror (a) and (b), respectively, but for normalized Vina scores.

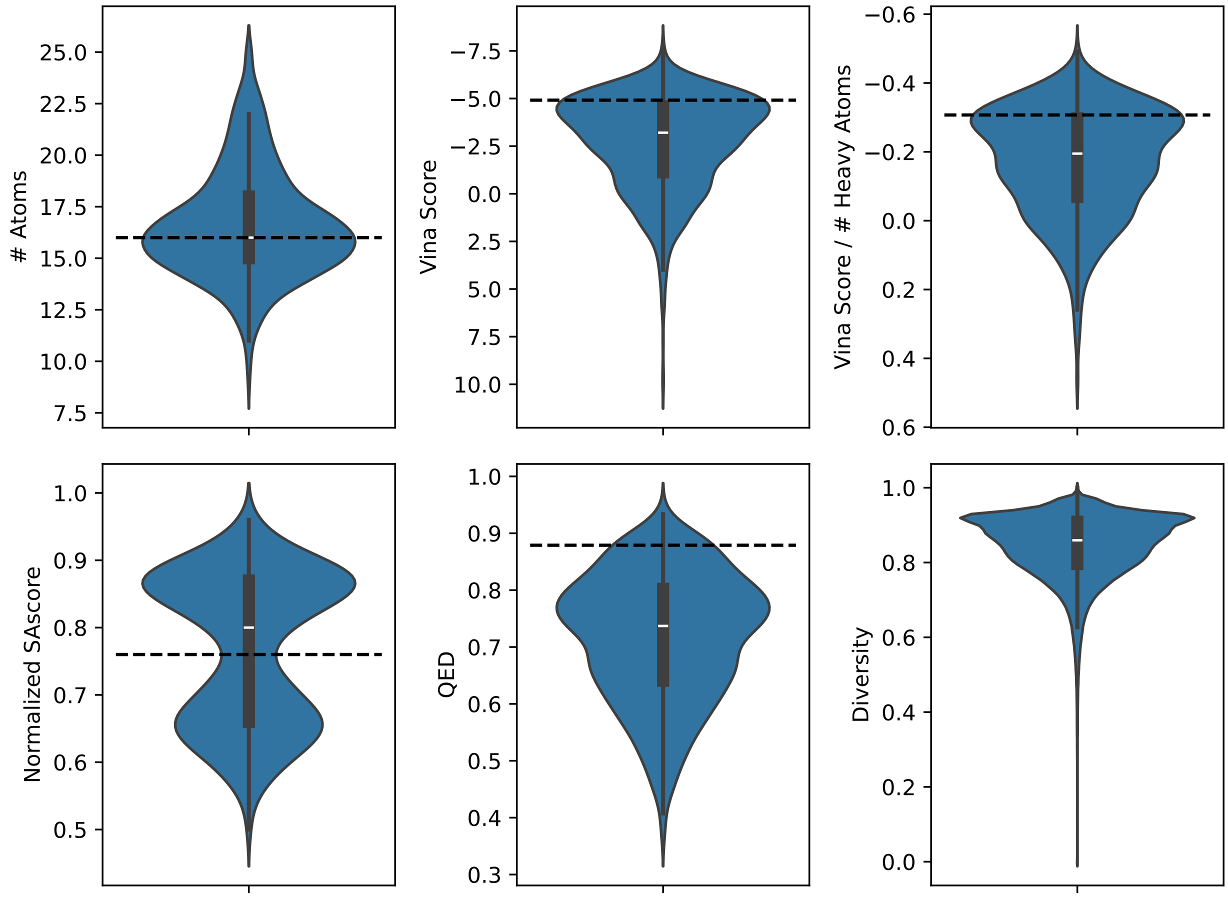

Figure S9: Distributions of properties of the 1139 unique molecules that passed all filters in Case Study 1. Distribution of the generated molecules in blue, the compound AtC-45 is shown with the dotted black line.

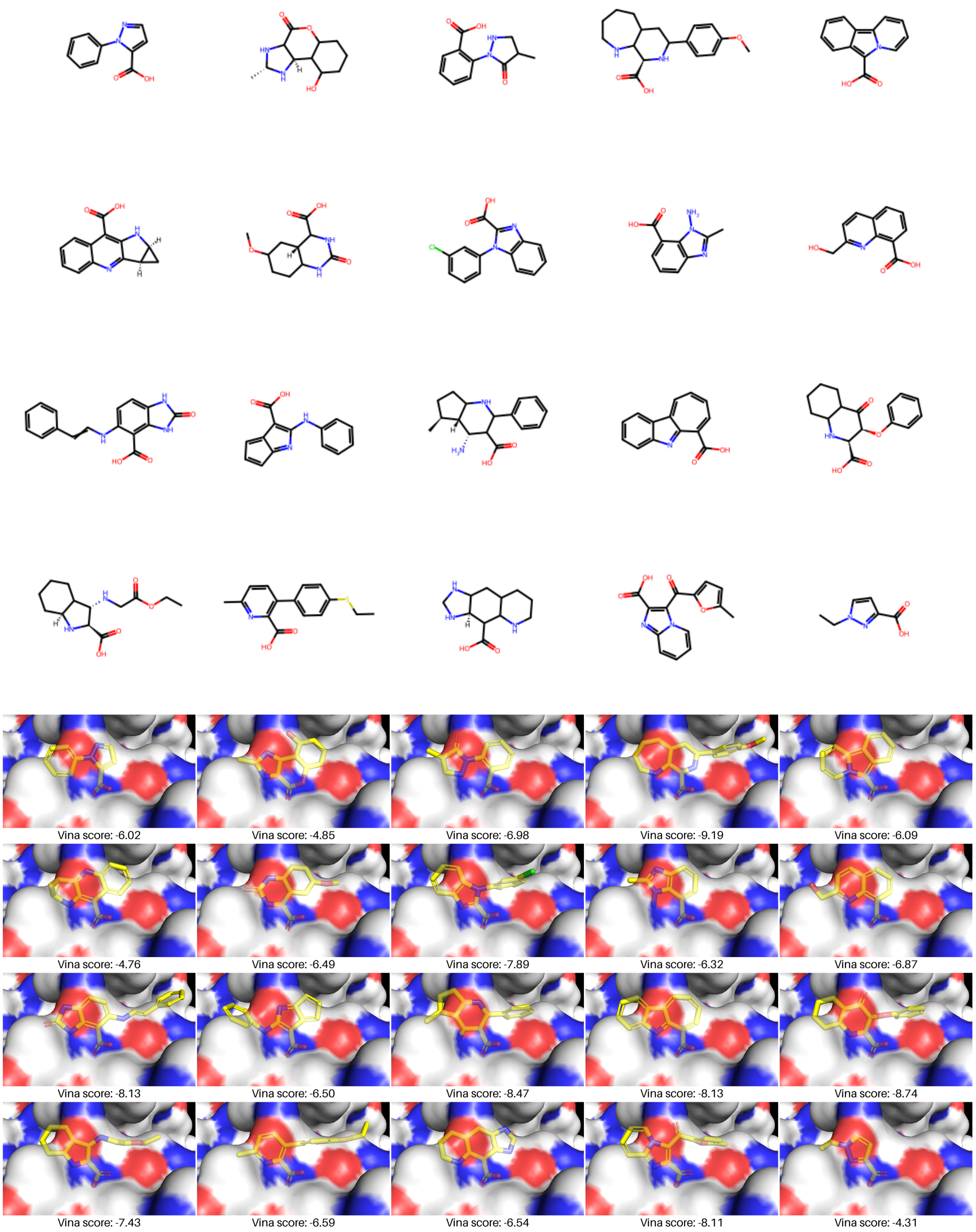

Figure S10: A random sample of the generated molecules from Case Study 1 shown as molecular structures (top) and in the binding site (bottom).

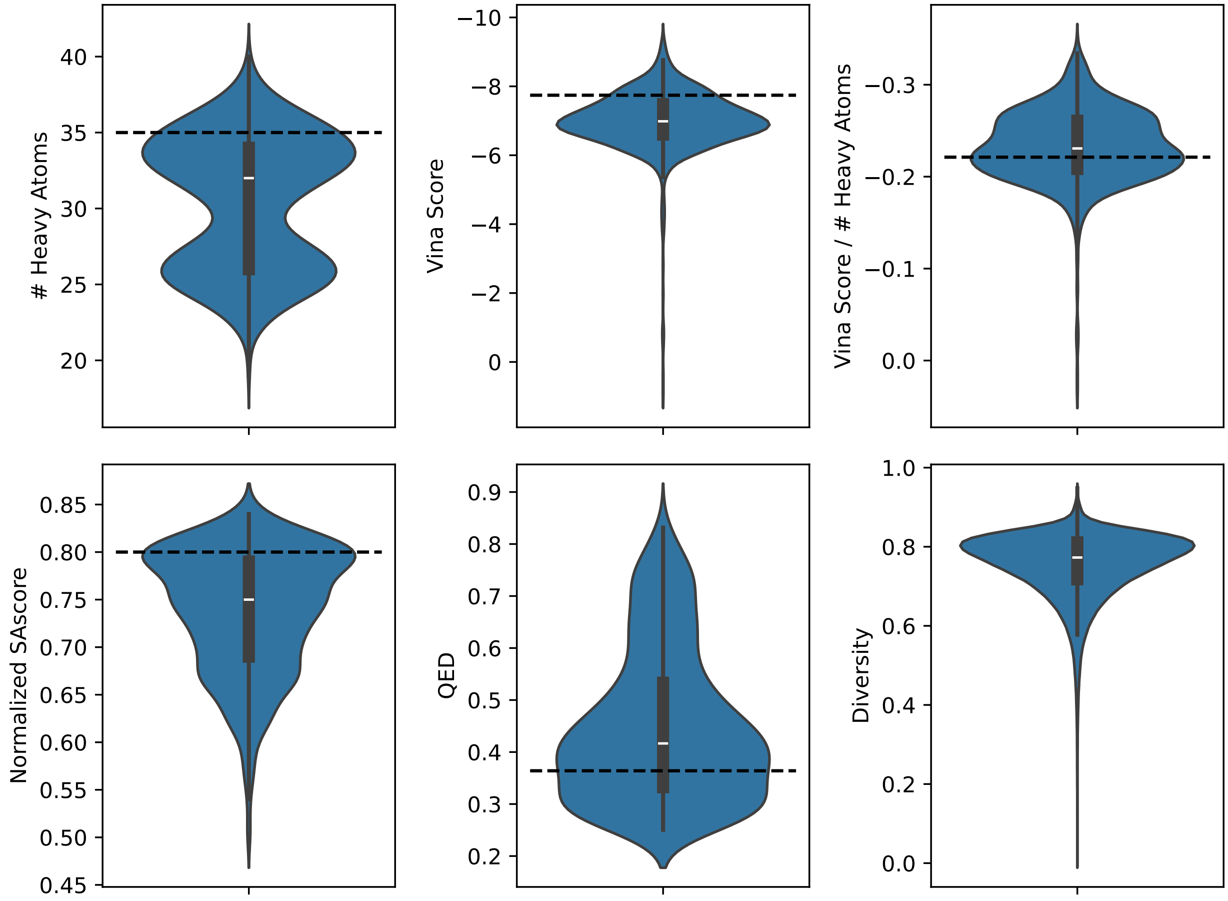

Figure S11: Distributions of properties of the 1077 unique molecules that passed all filters in Case Study 2. Distribution of the generated molecules in blue, Hit Compound 1 is shown with the dotted black line.

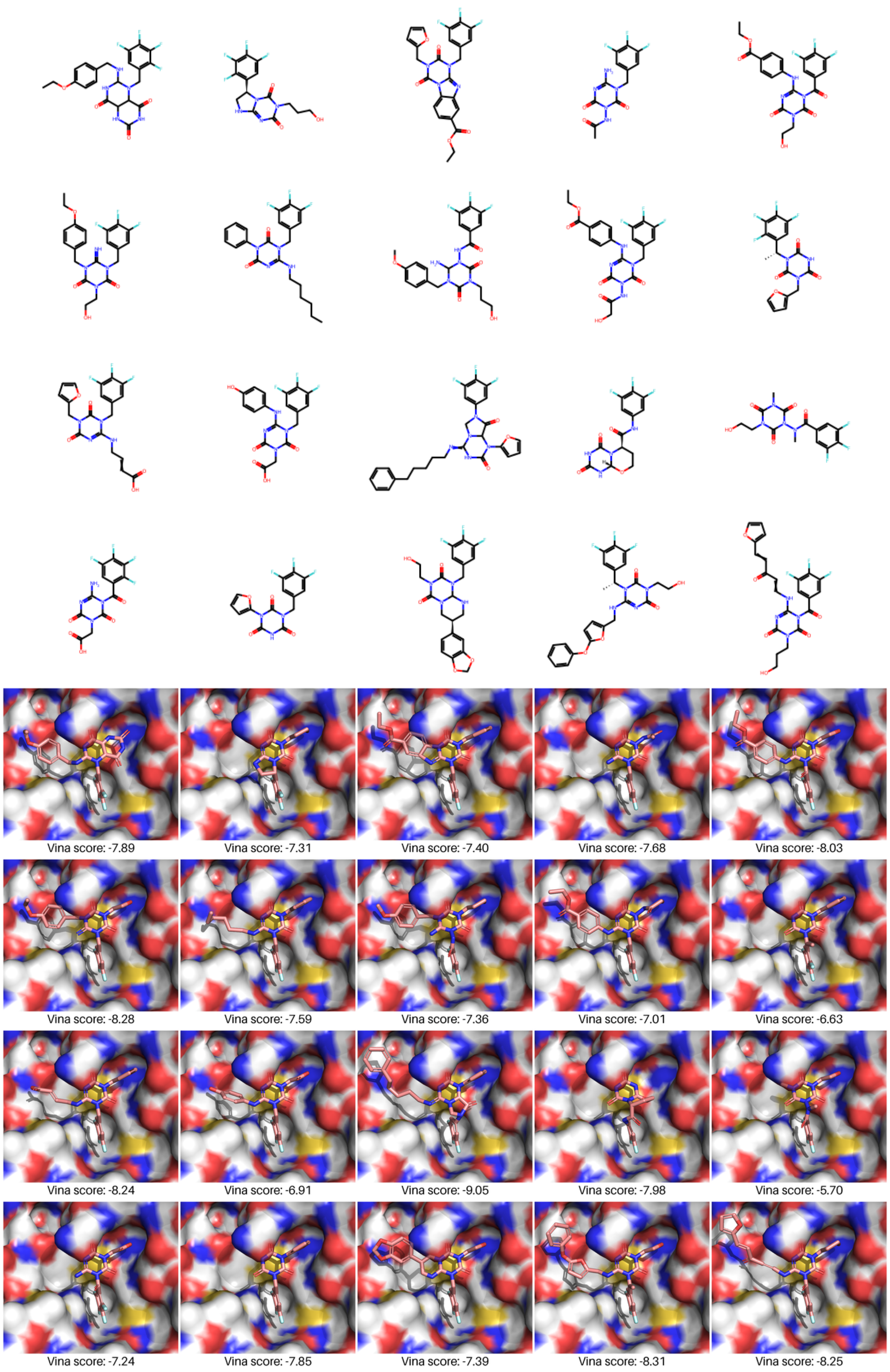

Figure S12: A random sample of the generated molecules from Case Study 2 shown as molecular structures (top) and in the binding site (bottom).

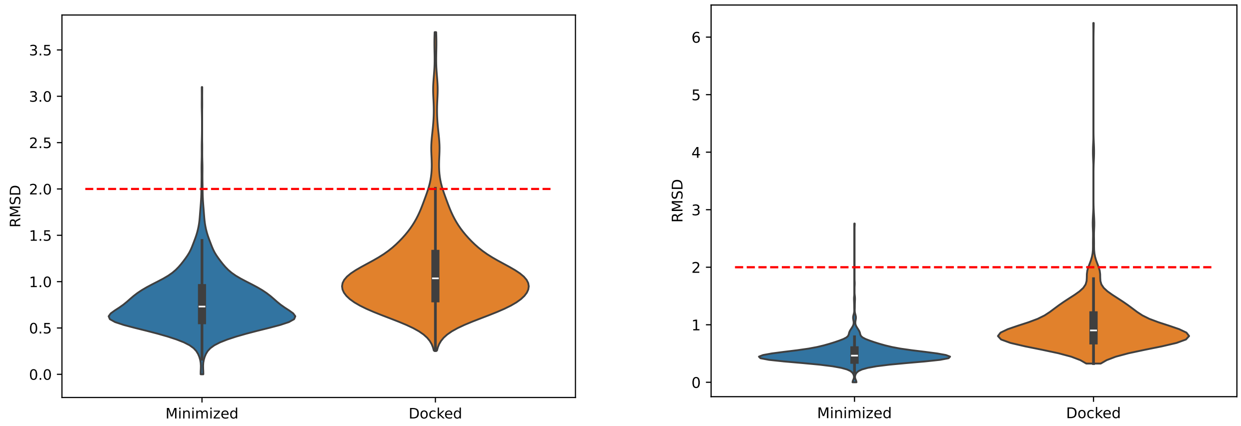

(a) (b)

Figure S13: RMSD upon minimization and redocking of (a) the 1139 unique molecules that passed all filters in Case Study 1 and (b) the 1077 unique molecules that passed all filters in Case Study 2. RMSD for redocking is the minimum RMSD across the ten generated poses. The mean RMSD was 1.13Å ± 0.48 and 0.99Å ± 0.42 for (a) and (b), respectively. Furthermore, 95% and 98% of generated molecules had a pose with RMSD below 2Å in (a) and (b), respectively.

References

(1) Eberhardt, J.; Santos-Martins, D.; Tillack, A. F.; Forli, S. AutoDock Vina 1.2. 0: New docking methods, expanded force field, and python bindings. *Journal of Chemical Information and Modeling* **2021**, *61*, 3891–3898.

(2) Weller, J. A.; Rohs, R. Structure-based drug design with a deep hierarchical generative model. *Journal of Chemical Information and Modeling* **2024**, *64*, 6450–6463.

(3) Guan, J.; Zhou, X.; Yang, Y.; Bao, Y.; Peng, J.; Ma, J.; Liu, Q.; Wang, L.; Gu, Q. DecompDiff: Diffusion Models with Decomposed Priors for Structure-Based Drug Design. *Proceedings of the 40th International Conference on Machine Learning* **2023**, *202*, 11827–11846.
